# Structural and Dynamic Insights into Acyl Carrier Protein upon Metal Binding and Acylation Revealed by NMR Spectroscopy and MD Simulations

**DOI:** 10.3390/ijms26189005

**Published:** 2025-09-16

**Authors:** Chae Yeong Lee, Sungchan Jang, Hyunjoon Cho, Min-Cheol Jeong, Yoojin Oh, Yangmee Kim

**Affiliations:** Department of Bioscience and Biotechnology, Konkuk University, Seoul 05029, Republic of Korea; chae022@konkuk.ac.kr (C.Y.L.); livin25@konkuk.ac.kr (S.J.); chogus99@konkuk.ac.kr (H.C.); mcjung91@gmail.com (M.-C.J.); milk6894@konkuk.ac.kr (Y.O.)

**Keywords:** NMR spectroscopy, acyl carrier protein, structure, dynamics, MD simulation, metal binding, acylation

## Abstract

Protein dynamics are crucial for the acyl carrier protein (ACP) acting as a cofactor, communicating with various fatty acid synthesis (FAS) enzymes. Using a combination of NMR spectroscopy and molecular dynamics (MD) simulations, we demonstrate how the conformational flexibility of *Escherichia coli* ACP (*Ec*ACP) modulates metal binding and facilitates its molecular switches, thereby determining the pathway for different acyl chains. Our results show that Ca^2+^ binding greatly stabilizes the protein—boosting thermal stability by over 13 °C—and modulates its dynamic properties, affecting two acidic metal binding sites and the conformation of the hydrophobic cavity. Hydrogen–deuterium exchange and chemical denaturation experiments revealed that Ile11 and Ile72 are the key residues for the global folding of *Ec*ACP, stabilizing hydrophobic cavity. Backbone dynamics and MD simulation results indicate that longer acyl chains induce conformational adjustments, increasing flexibility in α3-helix and hydrophobic motifs, including Phe28 and Ile54. Furthermore, our findings highlight the conformational plasticity of *Ec*ACP, with key molecular switches, Leu42 and Leu46, adapting to accommodate various acyl chains and directing their pathway. These insights deepen our understanding of ACP flexibility and its functional role in FAS, offering a new strategy for designing inhibitors that target the dynamic nature of bacterial FAS pathways.

## 1. Introduction

Acyl carrier proteins (ACPs) are essential cofactors that play a central role in a wide range of vital biochemical pathways, including fatty acid synthesis (FAS) [1], polyketide synthesis [2], and lipopolysaccharide biosynthesis [3]. Among these, FAS stands out as a fundamental process, not only forming the essential components of cellular membranes but also serving as a pivotal mediator in intracellular signaling and primary energy production [4]. ACPs are central to these pathways, serving as dynamic molecular shuttles that deliver growing acyl chains between enzymatic active sites. In type I FAS of fungi and mammalians, ACP is intricately integrated into a large, multifunctional megasynthase complex, where it orchestrates substrate transfer through covalent interactions, ensuring efficient chain elongation [5,6]. In contrast, in bacterial type II FAS, ACP exists as an independent protein that precisely delivers intermediates to individual enzymes, thereby enabling modular and adaptable fatty acid biosynthesis [7,8].

ACPs are small acidic proteins typically composed of four α-helices, with a molecular weight of approximately 10 kDa. The structures of type II FAS ACPs have been extensively studied [9,10,11,12,13,14], with *Escherichia coli* ACP (*Ec*ACP) serving as the most widely used model system [15,16,17]. A key feature of ACPs is the covalent attachment of fatty acid intermediates. Inactive apo-ACP is converted into the active holo form by holo-ACP synthase (ACPS), which attaches a 4′-phosphopantetheine (4′-PP) prosthetic group to the conserved serine residue within the DSL motif. The growing acyl chain is then covalently linked via a thioester bond to the 4′-PP arm of holo-ACP [18].

With the rising prevalence of multi-drug-resistant (MDR) Gram-negative bacteria, there is an urgent need for novel antibacterial targets. *E. coli* is a Gram-negative bacterium commonly found in the human lower intestine, where it exists as a versatile and mostly harmless commensal. However, the rapid spread of MDR strains within *E. coli* populations has made infections increasingly difficult and costly to treat. Especially, the emergence of MDR uropathogenic *E. coli* presents a serious threat that accelerates the demand for new antibiotic development. One promising strategy involves targeting proteins involved in FAS pathways, as these are essential for bacterial survival [19,20,21,22]. Recent research has therefore focused on investigating the structures and functions of FAS enzymes and ACPs [10,12,13,23,24,25,26,27,28]. Notably, ACP is highly dynamic, interacting with various partner proteins throughout the FAS process. Therefore, detailed studies of its structure, dynamics, and specific interactions with partner proteins are of significant interest for developing innovative antibacterial agents.

During communication with enzymes involved in fatty acid synthesis, the acyl chain flips back into the hydrophobic cavity of ACP for chain sequestration. This process, known as “chain-flipping”, has been described in previous studies [29,30]. When the elongating substrate is sequestered in the hydrophobic pocket, the highly dynamic and flexible α3-helix moves in the opposite direction to the α2-helix—its degree of movement depending on the acyl-chain length—to expand the pocket and accommodate the acyl chain substrate [1,29,31]. As the acyl-chain length increases, the cavity entrance—comprising the N-terminal region of the α2-helix, and the α3α4-loop—widens due to outward movement of the α3-helix, while the cavity bottom, including the C-terminal region of the α2-helix, the α2α3-loop, and the α1-helix, narrows to facilitate substrate sequestration [32]. Furthermore, the α3-helix, recognized as the most dynamic and structurally plastic region, serves as a gatekeeper in the “chain-flipping” process, undergoing conformational rearrangements that open a pathway to the partner enzyme’s active site, thereby enabling the acyl chain to flip out for transfer [29,33,34].

Bacterial ACPs possess numerous negatively charged residues, and fewer positively charged residues, which can lead to electrostatic repulsion. The “recognition α2-helix”, composed of highly conserved negatively charged residues, plays a crucial role in mediating interactions with other FAS enzymes. Previous studies have reported that *Ec*ACP contains two metal-binding sites: site A (comprising Glu30, Asp35, and Asp38) and site B (comprising Glu47, Asp51, Glu53, and Asp56) [35,36]. Furthermore, the addition of monovalent and divalent cations has been shown to increase the melting temperatures (T_m_) of ACPs [10,12,14,37,38], likely by neutralizing the electrostatic repulsion between negatively charged clusters on the surface of the protein. FAS enzymes often require divalent cation for their enzymatic activity. For instance, ACPS catalyzes the transfer of a 4′-PP group from coenzyme A to a serine residue on the apo-ACP, where Mg^2+^ plays an essential role in stabilizing the transition state and facilitating catalysis to create the active holo-ACP [39]. Therefore, investigating the interactions between FAS proteins and divalent cations is crucial for understanding the function and regulation of the FAS pathway.

The relationship between protein structure, dynamics, and function is highly complex and challenging to elucidate, primarily due to the extensive conformational space available to proteins and the wide range of timescales over which conformational motions occur. To effectively investigate these dynamic processes, combining solution NMR spectroscopy with molecular dynamics (MD) simulations has proven to be an invaluable approach. This synergistic methodology allows for a comprehensive exploration of protein motions at atomic resolution over various timescales, providing deeper insights into the structural flexibility and functional mechanisms of proteins [40,41,42].

Understanding the fundamental mechanisms underlying ACP stability and dynamics is critical for advancing the development of targeted antibacterial therapies. *Ec*ACP serves as a representative model system for bacterial ACP, and ongoing research into its structure and function remains highly relevant. In this study, we investigated how cationic metal ions can thermally stabilize *Ec*ACP and examined its chemical stability upon metal addition using guanidine hydrochloride (Gdn-HCl). Using NMR spectroscopy, we determined the high-resolution solution structure of holo-*Ec*ACP by incorporating residual dipolar coupling (RDC) data in the presence of Ca^2+^ ions and identified its Ca^2+^ binding sites. Hydrogen–deuterium (H/D) exchange and chemical denaturation experiments were employed to understand the key residues contributing to the global folding of the protein. Additionally, we explored the motional properties, molecular switching, and metal-binding characteristics through NMR spectroscopy and MD simulations of both holo- and various acylated forms, as well as differential scanning calorimetry (DSC) and circular dichroism (CD) experiments. Together, complementary techniques—NMR spectroscopy and MD simulations—serve as powerful tools that synergistically enhance our understanding of ACP’s structural and dynamic properties, particularly their interactions with metal ions and the effects of acylation. Our study provides valuable insights into the molecular mechanisms governing fatty acid synthesis regulation in bacteria.

## 2. Results

### 2.1. Comparative Sequence Analysis of EcACP with Mesophilic, Thermophilic, and Psychrophilic ACPs

**Figure 1:**
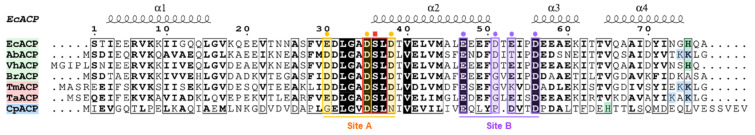
Sequence alignment of *Ec*ACP with mesophilic, thermophilic, and psychrophilic ACPs. Mesophilic ACPs are indicated with a green box, thermophilic ACPs with a red box, and psychrophilic ACPs with a blue box. The conserved DSL motif is highlighted in red, with the serine residue that covalently binds the 4′-phosphopantetheine (4′-PP) arm marked by a red square. The histidine and lysine residues are highlighted in green and blue, respectively. Two metal-binding sites are outlined in orange (site A) and purple (site B), with coordinating residues indicated by circles. *Ab*ACP refers to *Acinetobacter baumannii* ACP; *Vh*ACP to *Vibrio harveyi* ACP; *Br*ACP to *Brucella melitensis ACP*; *Tm*ACP to *Thermotoga maritima* ACP; *Ta*ACP to *Thermus aquaticus* ACP; and *Cp*ACP to *Colwellia psychrerythraea* ACP.

We compared the sequences of *Ec*ACP with mesophilic and thermophilic ACPs, and psychrophilic ACPs, highlighting key differences related to environmental adaptation. Sequence alignment reveals that the acidic residues in metal-binding sites A and B are highly conserved among all mesophilic ACPs (Figure 1). The DSL motif within metal-binding site A, including the 4′-PP attachment residue serine (Ser36 in *Ec*ACP), indicated by a red square, is fully conserved in all ACPs. Interestingly, Glu53 residue in site B of *Ec*ACP is replaced by lysine in the thermophilic *Thermotoga maritima* ACP (*Tm*ACP), which forms electrostatic interactions with nearby glutamate residues, contributing to greater structural stability and a higher T_m_ (Table 1) [13]. Comparison of the numbers of acidic and basic residues showed that thermophilic ACPs possess more basic residues (9–10), whereas mesophilic ACPs, including *Ec*ACP, have 5–6, and psychrophilic *Colwellia psychrerythraea* ACP (*Cp*ACP) contains only 2. This variation suggests adaptation mechanisms: increased basic residues in thermophiles stabilize the structure at high temperatures via salt bridges, while fewer basic residues in psychrophiles maintain flexibility at low temperatures for efficient enzyme interactions. Stacking interactions between side chains of histidine and tyrosine located at the α4-helix stabilize the helix, and this histidine may contribute to mitigating electrostatic repulsion, as the side chain maintains a positive charge at neutral pH [14]. Thermophilic ACPs, such as *Tm*ACP and *Thermus aquaticus* ACP (*Ta*ACP), have lysine residues instead of histidine residues at that position and often contain two lysines at their C-terminus. Comparison of T_m_ values among mesophilic ACPs showed that *Acinetobacter baumannii* ACP (*Ab*ACP), containing two lysines in its C-terminal region, exhibits a slightly higher T_m_ of 68.0 °C compared to *Ec*ACP (67.4 °C) and *Vibrio harveyi* ACP (*Vh*ACP) (66.4 °C), each with only one histidine. These findings suggest that the positive charges contributed by C-terminal histidine or lysine residues—located near the highly flexible N- and C-terminal regions—play a role in enhancing the structural stability of ACPs.

### 2.2. Thermal Stabilization of EcACP by Different Metal Ions

**Figure 2:**
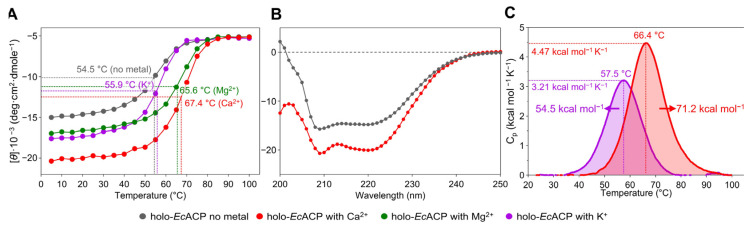
Thermal stability of *Ec*ACP as assessed by circular dichroism (CD) and differential scanning calorimetry (DSC) experiments in the presence of different metal ions. (**A**) Melting temperatures determined with no metal added (gray) and following the addition of Ca^2+^ (red), Mg^2+^ (green), and K^+^ (purple). (**B**) The effect of Ca^2+^ addition on the secondary structure of *Ec*ACP, compared between conditions without metal (gray) and with Ca^2+^ (red), as observed in CD experiments. (**C**) DSC transition curves showing the thermal profile in the presence of Ca^2+^ (red) and K^+^ (purple) with each maximum specific heat capacity (C_p_) and enthalpy values.

It is well-established that metal ion binding can mitigate the electrostatic repulsion in ACPs, thereby enhancing structural stability [14,45,46]. However, further investigation was necessary to examine how different metal ions influence the degree of stabilization. To this end, the thermal stability of *Ec*ACP was assessed under conditions without metal ions and with K^+^, Mg^2+^, or Ca^2+^ added, using circular dichroism (CD) experiments (Figure 2A). In the absence of metal ions, the T_m_ was 54.5 °C, which was the lowest among all tested conditions. The addition of K^+^ resulted in a slight increase in T_m_ to 55.9 °C, but this was significantly lower compared to divalent cations such as Mg^2+^ and Ca^2+^. When Mg^2+^ and Ca^2+^ were introduced, the T_m_ values increased substantially, reaching 65.6 °C with Mg^2+^ and 67.4 °C with Ca^2+^. Both conditions showed an increase of over 10 °C, conclusively demonstrating that divalent cations more effectively mitigate electrostatic repulsion and stabilize *Ec*ACP. The α-helix structure exhibits double minima characteristics at 208 nm and 222 nm, indicative of well-preserved α-helical secondary structure. Notably, when comparing these CD spectra, the intensity of these minima increased in the presence of Ca^2+^ (Figure 2B), suggesting an increase in ellipticity and implying a more stable, folded conformation of the protein in the presence of this divalent cation.

DSC experiments were conducted to assess the contribution of divalent (Ca^2+^) and monovalent (K^+^) cation to the structural stability, by comparing its thermal stability in the presence of these ions (Figure 2C). The T_m_ measured by DSC was 66.4 °C with Ca^2+^ and 57.5 °C with K^+^ (Figure 2C). The transition occurred at a significantly higher temperature with Ca^2+^, a finding consistent with the CD results (Figure 2A). Additionally, the maximum specific heat capacity (C_p_) in the presence of Ca^2+^ was 4.47 kcal mol^−1^ K^−1^, compared to 3.21 kcal mol^−1^ K^−1^ with K^+^, indicating a difference of 1.26 kcal mol^−1^ K^−1^ (Figure 2C). The calorimetric enthalpy (ΔH_cal_) obtained from the total area under the transition curve, was 71.2 kcal mol^−1^ with Ca^2+^, which is 16.7 kcal mol^−1^ higher than the 54.5 kcal mol^−1^ measured with K^+^. These results suggest that divalent cations, like Ca^2+^, help maintain a more compact hydrophobic core, thereby further stabilizing the structure.

### 2.3. NMR Structure of EcACP in the Presence of Ca^2+^

**Figure 3:**
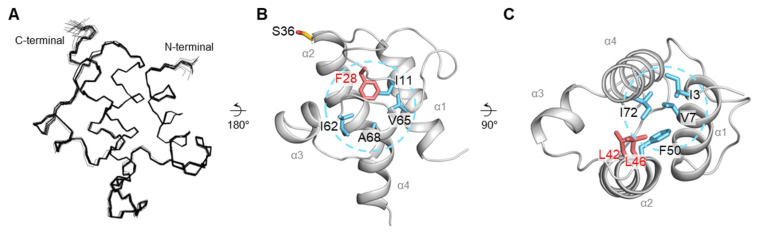
Solution structure of holo-*Ec*ACP in the presence of Ca^2+^ (PDB ID 9WAB). (**A**) Superimposition of the backbone atoms from the 20 lowest-energy structures of holo-*Ec*ACP, illustrating structural convergence. (**B**) The 4′-PP attachment residue, Ser36, is colored yellow. Position of Phe28 at the entrance of the cavity, highlighted in pink, is deeply embedded within the hydrophobic cavity, where it strengthens interactions with hydrophobic residues Ile11, Ile62, Val65, and Ala68, indicated by sky-blue dashed circle. (**C**) The hydrophobic triad, formed by Ile3, Phe50, and Ile72, along with Val7 at the top end of the cavity, is highlighted in sky-blue. Switch resides, Leu42 and Leu46, are colored in pink.

Although several structures *Ec*ACP and related studies have significantly advanced our understanding of its structural features and conformational changes due to metal ion binding, as well as acylation and interactions with other partner enzymes, high-resolution information on the solution structure of the holo form of *Ec*ACP interacting with divalent cation remains limited. To date, the crystal structure of holo-*Ec*ACP has only been obtained in complex with ACPS (PDB ID 5VCB), and stand-alone X-ray model of holo-*Ec*ACP has not been reported. Solution NMR studies have provided valuable insights into the solution structure of holo-*Ec*ACP (e.g., PDB IDs 1ACP and 2K93) in potassium phosphate buffer. In this study, we determined the high-resolution solution structure of holo-*Ec*ACP in the presence of Ca^2+^, incorporating RDC data, to achieve a well-defined structure of the protein bound to a divalent metal ion. The backbone and heavy-atom root-mean-square deviations (RMSDs) for the 20 lowest-energy models are 0.20 Å and 0.50 Å, respectively, indicating good convergence (Figure 3A and Table S1). The overall solution structure of *Ec*ACP consists of four α-helices—α1 (3–15), α2 (36–50), α3 (56–61), and α4 (65–74)—connected by three loops.

To understand how Ca^2+^ ions stabilize *Ec*ACP, we examined its structure focusing on previously identified metal-binding sites [35]. At metal binding site A, composed of Glu30, Asp35, and Asp38, Ca^2+^ binding stabilizes the long α1α2-loop, promoting an inward conformation compared with crystal structure (PDB ID: 1L0I). The phenyl ring of conserved Phe28 to be deeply embedded within the hydrophobic cavity, enhancing interactions with other hydrophobic residues (Figure 3B). As Phe28 is located near the cavity entrance, where it likely interacts with the incoming acyl chain and may facilitate its proper accommodation via subtle reorientation of its phenyl ring plane [47].

In the bottom region of the cavity, a conserved hydrophobic triad, composed of Ile3, Phe50, and Ile72, along with Val7, creates a hydrophobic patch that can interact with the tail of longer acyl chains (Figure 3C). Additionally, Leu42 and Leu46 in the α2-helix function as switch residues, as their side chain rotations regulate the path that the acyl chain follows within the cavity (Figure 3C) [47].

### 2.4. Structural Stability Analysis Through Hydrogen–Deuterium Exchange and Chemical Denaturation Experiments

**Figure 4:**
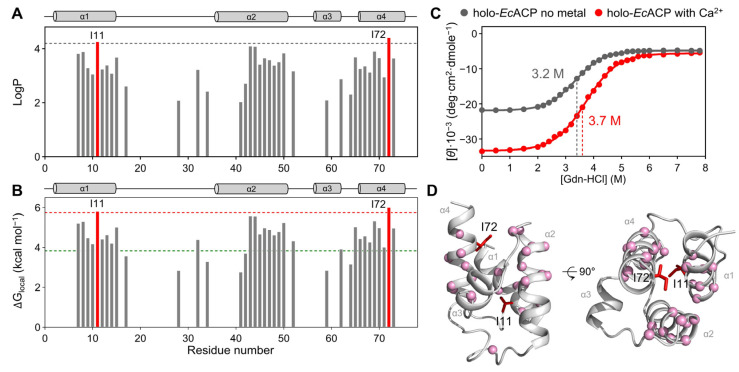
Hydrogen–deuterium exchange and chemical denaturation experiments on *Ec*ACP. (**A**) LogP value for each residue of *Ec*ACP in presence of Ca^2+^. The gray dashed line denotes LogP = 4.2. (**B**) The local unfolding free energies (ΔG_local_) for each residue of *Ec*ACP in presence of Ca^2+^. The green dashed line indicates the global unfolding free energy (ΔG_global_) of *Ec*ACP. The red dashed line represents 1.5 × ΔG_global_, which was used as a reference threshold. (**C**) Denaturation curves of *Ec*ACP measured with guanidine hydrochloride (Gdn-HCl) comparing conditions without metal (gray) and with Ca^2+^ (red). (**D**) Residues with ΔG_local_ values higher than ΔG_global_ values are represented as pink spheres in the solution structure of *Ec*ACP. Ile11 and Ile72 are highlighted in red.

To further investigate the stability of structural packing of holo-*Ec*ACP in its stable state in the presence of Ca^2+^, we performed an H/D exchange experiment on the backbone amide protons (Figure 4A,B). This technique analyzes the exchange rate of amide hydrogen within the protein structure, enabling assessment of structural stability and flexibility on timescales ranging from milliseconds to seconds or even longer. Protection factors (LogP), which are calculated through comparison of experimentally measured H/D exchange rate (k_prot_) with the random coil exchange rate (k_rc_), were employed for each residue to quantitatively evaluate the degree to which the amide protons were protected from exchanging with deuterium in solution (Figure 4A).

In holo-*Ec*ACP, all α-helical regions except α3-helix exhibited high LogP values, indicating that the α1-, α2-, and α4-helices are relatively stable. Within the α3-helix, only Ala59 showed slow amide proton exchange with deuterium, whereas in apo-*Ec*ACP, slow amide proton exchange was observed for all residues of α3-helix [48]. This suggests that the transition from the apo to the holo form specifically affects the stability of the α3-helix, likely due to the movement of the prosthetic group. Ala59 maintains a high amide proton protection because its side chain faces the hydrophobic pocket rather than the solvent, thus minimizing external interactions and stabilizing its backbone amide proton. Notably, even after 1000 min, amide protons of residues such as Ile11, Val43, Ile46, Glu47, Ile69, and Ile72 remained, reflecting persistent structural stability in these regions over time. Phe28 in the α1α2-loop, responsible for the proper accommodation of acyl chain at the entrance of cavity, also exhibited a high LogP value, likely because its phenyl ring interacts with other hydrophobic residues within the cavity, thereby contributing to a more compact structural packing. Leu42 and Leu46, which function to determine the path of the acyl chain, also showed protection of the amide proton. In addition, to analyze the local unfolding, we calculated the free energies of local unfolding (ΔG_local_) for each residue based on H/D exchange data (Figure 4B). A consistent trend with the LogP values was observed: residues in helical regions, except for the α3-helix, exhibited greater stability.

To assess how the presence or absence of Ca^2+^ affects the chemical stability of *Ec*ACP and to compare the free energies of global unfolding (ΔG_global_) with ΔG_local_, we conducted chemical denaturation experiments using Gdn-HCl. The half-denaturation concentration ([Gdn-HCl]_1_/_2_)—the concentration at which half of the protein structure begins to denature—was measured for *Ec*ACP (Figure 4C). In the presence of Ca^2+^, [Gdn-HCl]_1/2_ was 3.7 M whereas in the absence of metal ions, it decreased to 3.2 M. This indicates that metal ions enhance not only the thermal stability but also the chemical stability of *Ec*ACP, promoting a more stable folded conformation.

Using the denaturation curves of chemical denaturation experiments, we calculated the ΔG_global_. The ΔG_global_ of *Ec*ACP in the presence of Ca^2+^ was determined to be 3.83 kcal mol^−1^ at [Gdn-HCl]_1/2_. Comparing the ΔG_local_ from H/D exchange experiments with the ΔG_global_, we identified residues with a higher ΔG_local_ than ΔG_global_: Val7, Lys8, Lys9, Ile10, Ile11, Gly12, Glu13, Gln14, Leu15, Leu32, Val43, Met44, Ala45, Leu46, Glu47, Glu48, Glu49, Phe50, Thr52, Ile62, Val65, Gln66, Ala67, Ala68, Ile69, Asp70, Tyr71, Ile72, and Asn73. These residues are predominantly located in the α-helix, and they are indicated by pink spheres in Figure 4D.

The Ile11 in the α1-helix, and the Ile72 in the α4-helix showed the highest ΔG_local_ values (5.82 kcal mol^−1^ for Ile11 and 6.01 kcal mol^−1^ for Ile72), which were more than 1.5 times higher than that of ΔG_global_, indicating that these residues are the critical for protein folding. These residues are conserved across multiple ACP homologs, underscoring their structural and functional importance (Figure 1). The Ile11 of the α1-helix is a key residue contributing to the formation of a hydrophobic core, and it also maintains the tight packing of the structure. Also, Ile72 stabilizes the structure of *Ec*ACP by interacting with other hydrophobic residues, such as Ile3 and Phe50, at the top end of the hydrophobic cavity. As these two residues dominate hydrophobic interactions, residues such as Val7, Leu46, Phe50, Ile62, and Ala68 also exhibited higher ΔG_local_ values than the ΔG_global_ value. Ile11 and Ile72 are also represented in the structure of *Ec*ACP in Figure 4D.

### 2.5. Analysis of Metal-Binding Sites by Metal Titration HSQC Spectra

**Figure 5:**
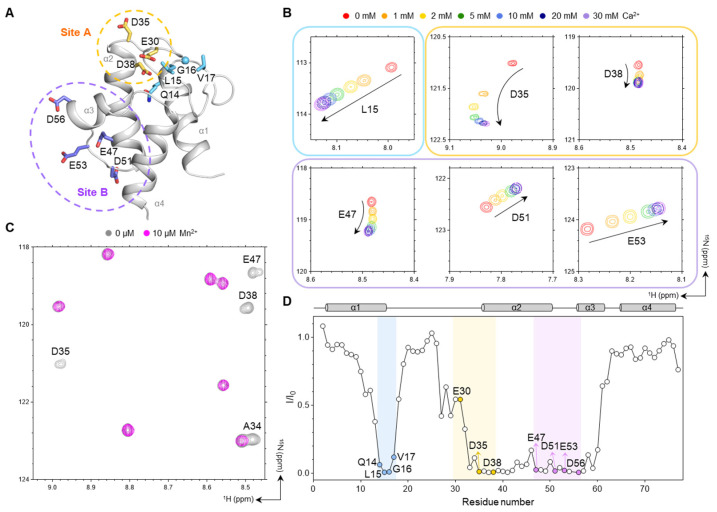
Metal titration experiments of *Ec*ACP. (**A**) Structural representation of metal binding sites, with site A highlighted in yellow, and site B in purple. Residues near site A are highlighted in sky-blue. (**B**) ^1^H-^15^N HSQC spectra showing peak traces of residues within the metal-binding sites during titration with Ca^2+^ at various concentrations: 0 mM (red), 1 mM (orange), 2 mM (yellow), 5 mM (green), 10 mM (blue), 20 mM (navy), and 30 mM (purple). (**C**) Peak disappearance or broadening in the spectra in the absence (gray) and presence of 10 µM Mn^2+^ (magenta). (**D**) Quantitative plot illustrating the ratio of peak intensities of residues in the presence of 10 µM Mn^2+^ relative to no Mn^2+^, indicating the proximity of these residues to the paramagnetic Mn^2+^. Metal-binding sites A and B and residues near site A (Gln14, Leu15, Gly16, and Val17) are highlighted in yellow, purple, and sky-blue, respectively.

Previous studies have identified two metal-binding sites in *Ec*ACP: Site A, comprising Glu30, Asp35, and Asp38; and Site B, composed of Glu47, Asp51, Glu53, and Asp56 (Figure 5A) [35,36]. In our Ca^2+^ titration experiments with concentrations ranging from 0 to 30 mM, both sites showed chemical-shift perturbations (CSPs), with the magnitude increasing at higher Ca^2+^ concentrations (Figure 5B and Figure S1A). As Ca^2+^ is a diamagnetic ion, it perturbs the local structure showing CSPs in the ^1^H–^15^N heteronuclear single-quantum coherence (HSQC) spectra. As shown in Figure 5B, these residues near the metal-binding sites exhibited significant CSPs during titration. In addition to the residues directly involved in metal binding, residues located near site A, such as Leu15, exhibited significant CSPs (Figure 5B). Interestingly, Ile54, located between the site B residues, exhibited the largest CSP, despite not directly coordinating the Ca^2+^. This suggests that Ca^2+^ binding to site B induces substantial structural rearrangement of the loop region, especially Ile54, thereby stabilizing its conformation (Figure S1B,C).

To assess the proximity of residues to these metal-binding sites, additional titration experiments using Mn^2+^—a strongly paramagnetic ion—were performed (Figure 5C and Figure S2). Even at low concentrations (10 μM), Mn^2+^ induced severe line broadening, resulting in significant intensity reduction for residues such as Glu30 or rapid disappearance of peaks for Asp35, Asp38, Glu47, Asp51, Glu53, and Asp56 from sites A and B (Figure 5C,D), confirming their close proximity to bound metal ions. Residues near site A (Gln14, Leu15, Gly16, and Val17), which also showed CSPs in the Ca^2+^ titration, displayed marked signal loss, indicating that even hydrophobic residues not directly coordinating the metal can undergo structural rearrangement when located adjacent to site A, as the region is stabilized upon metal binding (Figure 5A,D).

### 2.6. Chemical Shift Perturbation by Acylation of EcACP

**Figure 6:**
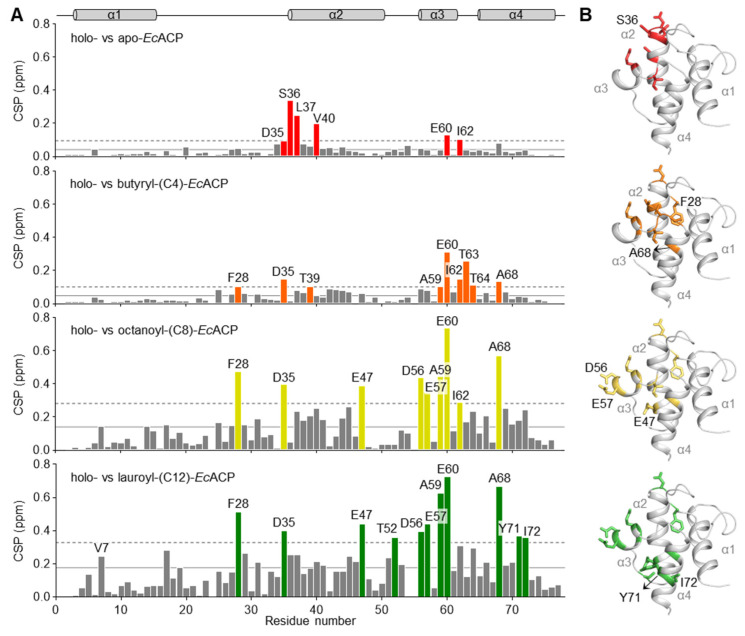
Chemical-shift perturbation analysis of acylated *Ec*ACP. (**A**) Bar graph showing the chemical shift perturbation (CSP) changes between the acylated forms and holo form of *Ec*ACP. The solid lines represent the average value, and the dotted lines above represent the average value plus one standard deviation. Residues with CSP values exceeding the average plus-one standard deviation are highlighted in color. (**B**) Structural representation illustrating these residues with CSPs highlighted in their respective colors, as shown in (**A**).

To assess structural perturbations caused by different acyl-chain lengths, we acquired and compared ^1^H–^15^N HSQC spectra for various acylated forms and analyzed the corresponding CSPs (Figure 6). The transition from the apo to holo form, with the 4′-PP attachment to Ser in the DSL motif, resulted in large CSPs in residues nearby this motif. Glu60 and Ile62, located at the cavity entrance, also exhibited significant CSPs, suggesting that the α3-helix and α3α4-loop not only participate in cavity expansion but are also affected by interactions between the 4′-PP arm and this region [31,49].

A significant CSP between the holo and butyryl form was newly observed for a key residue, Phe28, which is located near the cavity entrance. Phe28 is likely perturbed upon insertion of the butyryl group, indicating a direct interaction. Additionally, a notable CSP was observed for Ala68, located at the N-terminal region of the α4-helix and oriented toward the cavity interior, whereas residues at the bottom of the cavity exhibited minimal CSPs. These findings suggest that the short butyryl group can be accommodated only up to the midpoint of the cavity. In addition, residues Glu57, Ile54, and Asp56 at α3-helix exhibited minimal CSPs. These residues are located within or near metal-binding site B, and the reduced CSP values are presumably caused by structural stabilization upon Ca^2+^ binding, leading to comparatively decreased flexibility of this region in the short butyryl form.

Upon transition to the octanoyl form, residues in the α3-helix, including Asp56 and Glu57, exhibited significantly increased CSPs, indicating conformational rearrangement in the α3-helix associated with the accommodation of the longer acyl chain-like octanoyl group. In addition, increased CSP values were observed for residues in the α2-helix, particularly around Glu47, when comparing CSPs between the holo and butyryl forms. These observations suggest that the cavity undergoes a more pronounced expansion in the octanoyl form than in the butyryl form, primarily involving the rearrangement of α3-helix, with the α2-helix likely affected by structural adjustments.

In the lauroyl form, CSPs of Tyr71 and Ile72 in the α4-helix increased significantly. The large CSP values observed for these residues at the cavity bottom are consistent with previous studies showing hydrophobic contacts between the longer acyl chain and these residues [17,50]. Overall, these findings highlight that the extent and pattern of CSPs are dependent on acyl-chain length, with longer chains inducing more pronounced structural rearrangements in the α3-helix and additional perturbations within the cavity, reflecting progressive cavity adaptation. In the case of longer chains, such as the palmitoyl form, hydrolysis occurs so rapidly that the percentage of the holo form far exceeds that of the acylated form, making it impossible to analyze the CSPs.

### 2.7. Backbone Dynamics of Holo-, Butyryl-, and Octanoyl-EcACP

**Figure 7:**
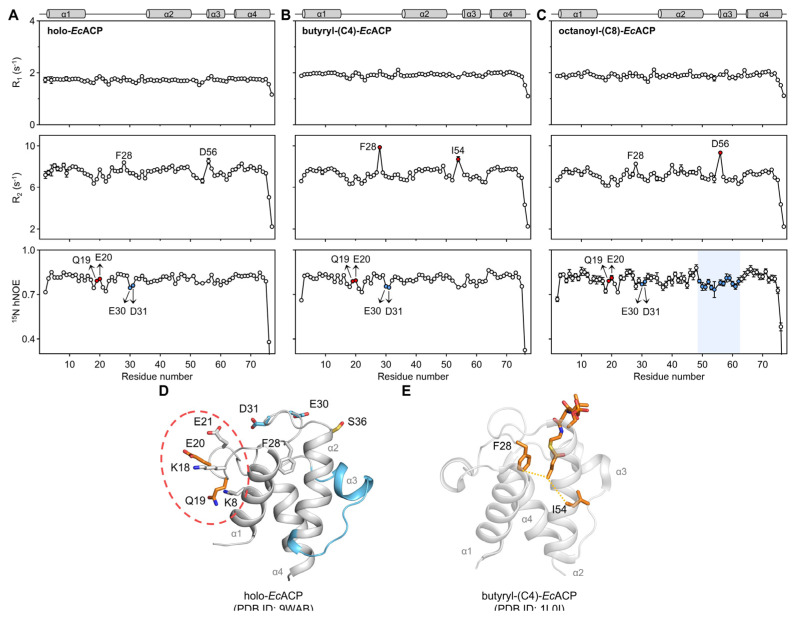
Backbone dynamics of *Ec*ACP. ^15^N-longitudinal relaxation (R_1_), transverse relaxation (R_2_), and ^1^H-^15^N heteronuclear NOE (hNOE) values for (**A**) holo-, (**B**) butyryl-, and (**C**) octanoyl-*Ec*ACP. Residues with high R_2_ or hNOE values are marked in red, and those with low hNOE values in sky blue. (**D**) Residues Glu30 and Asp31, located at the cavity entrance and regions with low hNOE values in the octanoyl form, are highlighted in sky blue. In contrast, Gln19 and Glu20, which show high hNOE values and are positioned to potentially form salt bridges with nearby polar residues, are marked with a red dashed circle. (**E**) Residues exhibiting high R_2_ rates, such as Phe28 and Ile54—resulting from their contact with the butyryl chain—are identified in the crystal structure of butyryl-*Ec*ACP (PDB ID: 1L0I). All experiments were performed at 700 MHz and 25 °C.

To characterize and compare the backbone dynamics of *Ec*ACP according to various chain length on a ps-ns timescale, we measured the spin-relaxation rates of longitudinal (R_1_) and transverse (R_2_) and heteronuclear NOEs (hNOEs) for the holo, butyryl, and octanoyl forms of *Ec*ACP (Figure 7A–C).

Across all three forms, Gln19 and Glu20 consistently displayed relatively high hNOE values despite being located in the α1α2-loop. This may reflect enhanced structural stability resulting from the formation of salt bridges with nearby polar residues (Figure 7D).

In the butyryl form, Phe28 in the α1α2-loop, and Ile54 in the α2-helix exhibited significantly higher R_2_ rates (9.86 and 8.72 s^−1^, respectively), likely reflecting interactions between the terminal end of the butyryl chain and the side chains of Phe28 and Ile54 with close spatial proximity, both oriented toward the cavity interior (Figure 7E). In contrast, in the holo form, the 4′-PP arm is too short to influence these residues much, while in the octanoyl form, the longer chain remains stably accommodated within the cavity. Due to these differences in chain length, significant exchange was observed only for butyryl form at these residues. The previously reported crystal structure of butyryl-*Ec*ACP (PDB ID 1L0I) is consistent with this interaction pattern [51] (Figure 7E). In contrast, these residues exhibited relatively low R_2_ rates in the holo and octanoyl forms. In the holo form, the acyl chain is too short to interact with these residues, while in the octanoyl form, the chain is more stably embedded within the cavity, resulting in less pronounced exchange compared to the butyryl form.

In the octanoyl form, Asp56, located in the N-terminal region of the α3-helix, exhibited a significantly higher R_2_ rate compared to the holo and butyryl forms. This suggests that accommodation of the octanoyl chain involves movement of the α3-helix, which is supported by low hNOE values, reflecting increased flexibility in this region (Figure 7C). Moreover, from the holo to the butyryl and octanoyl forms, hNOE values in the α3-helix and adjacent loop regions progressively decrease, indicating that longer acyl chains are accompanied by greater outward movement of α3-helix to accommodate them.

Glu30 and Asp31, located in the α1α2-loop, exhibited notably lower hNOE values compared to near residues in the loop region (Figure 7D). This increased flexibility is likely due to their position at the cavity entrance, where they are solvent-exposed, as well as the dynamic motion of Phe28, which forms transient interactions with short acyl chains, such as the butyryl chain. Although Ca^2+^ binding may enhance the overall stability of the loop, Glu30 and Asp31 at the cavity entrance are functionally required to retain conformational flexibility. Furthermore, hNOE values of Glu30 and Asp31 slightly increased in the octanoyl form compared to holo and butyryl forms, suggesting that the longer acyl chain stabilizes the cavity entrance, reflecting reduced dynamic fluctuations at the cavity entrance due to tighter acyl-chain packing.

### 2.8. Molecular Dynamics Simulation of EcACP

**Figure 8:**
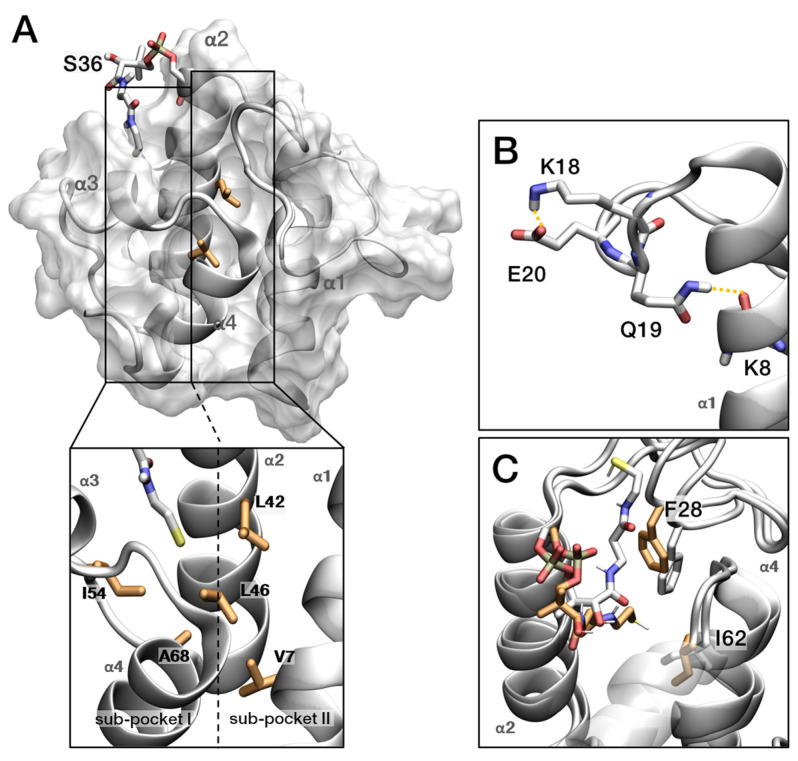
MD simulations on holo-*Ec*ACP showing the movement of the key residues. (**A**) Detailed illustration of holo-*Ec*ACP. Inside the cavity, sub-pocket I and sub-pocket II are separated by two leucine residues of α2 helix (dashed line). Hydrophobic residues constituting the pocket are depicted in orange. (**B**) Hydrogen bond distance between Gln19 side chain (NE) and the backbone carbonyl oxygen of Lys8. Additional hydrogen bonds between Lys18 and Glu20 are also depicted. (**C**) Phe28 and Ile62 tilting related to the entry of the prosthetic linker in holo-*Ec*ACP.

To investigate structural movements of *Ec*ACP during acyl-chain accommodation, we performed 1000 ns MD simulation on holo-, butyryl-, octanoyl-, lauroyl-, and palmitoyl-*Ec*ACP. The acyl chain and prosthetic group were initially solvent-exposed, allowing us to observe their entry into the hydrophobic cavity. First, the residues that exhibited significant structural changes are examined within the holo-*Ec*ACP structure. *Ec*ACP features a 4′-PP arm that binds acyl intermediates from the Type II-FAS pathway, with its internal cavity divided into two separate sub-pockets designed to accommodate a wide range of acyl-chain lengths (Figure 8A). Additionally, hydrophobic residues such as Ile54, Ala68, and Val7 line the interior, forming a hydrophobic environment facing inward. Leu42 and Leu46 are aligned along the α2-helix and positioned between sub-pocket I and sub-pocket II, contributing to the structural framework necessary for substrate binding and accommodation.

The residue from 19 to 21 forms hydrogen bonds, despite residing in the α1α2-loop, and these bonds display relatively high hNOE values (Figure 8B). During the 1 μs MD simulations, we observed the sidechain of Glu19 and backbone of Lys8 retain stable hydrogen bonding within 3.5Å about 40% occupancy, and this proximity is further supported by observed bending and fluctuations in this loop region. In addition, loop bending enables Lys18 to access the side chain of Glu20, forming an extra hydrogen bond occasionally (~5% occupancy). Two types of hydrogen bonds strengthen the interaction between the α1-helix and the α1α2-loop, influencing the backbone dynamics of *Ec*ACP.

Ile62 changes its torsion angle corresponding to the entry of the 4′-PP arm, suggesting that Ile62 may mirror structural changes associated with variations in acyl-chain length (Figure 8C). Since Ile62 has a significant value of CSP in holo- and octanoyl-*Ec*ACP, we compared it with the distance data, which are the extent to which the acyl chain enters the cavity, and the torsion angle value of the Ile62 side chain in octanoyl-*Ec*ACP (Figure S3A). We observed that when the side chain rotates by approximately −70 degrees, the eight carbon molecules became deeply sequestered, suggesting that Ile62 is a residue that responds to acyl-chain entry.

**Figure 9:**
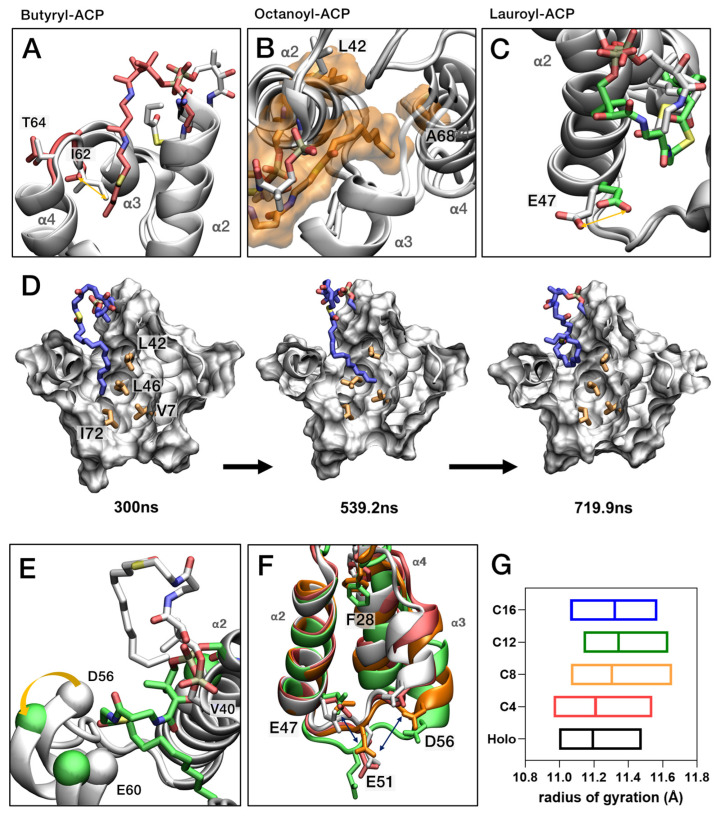
Molecular dynamics simulation results of *Ec*ACP with various acyl chains. (**A**–**C**) Structural changes in *Ec*ACP with increasing acyl-chain length from holo-*Ec*ACP: (**A**) butyryl-*Ec*ACP, (**B**) octanoyl–*Ec*ACP, and (**C**) lauroyl-*Ec*ACP. The transparent shading illustrates the molecular surface using the VMD Surf drawing method. (**D**) Cavity of palmitoyl-*Ec*ACP, illustrating the entry of the acyl chain from sub-pocket I to sub-pocket II at 300 ns, 539.2 ns, and 719.9 ns. (**E**) Outward movement with the increasing distance from Val40, indicating cavity expansion for acyl-chain entry. (**F**) Fluctuations in residues with negative charges near the α2α3-loop, reflecting shifts in Glu60 that suggest electrostatic adjustments in response to longer acyl chains. (**G**) Radius of gyration from each model, suggesting overall swelling of the protein structure with increasing acyl-chain length.

To understand the fatty acids biosynthesis mechanism, we investigated how the structure of *Ec*ACP adapts to varying substrate lengths by modeling butyryl-, octanoyl-, lauroyl-, and palmitoyl-*Ec*ACP, each extending the acyl chain by four carbon units. Butyryl-*Ec*ACP serves as the initial comparison with holo-*Ec*ACP, representing the effect of a short acyl chain. Due to its short acyl chain, it was insufficient to sample a stable conformation or sustain long-term (around 200 ns) sequestration. Similar dynamics were observed between the Ile62 switch pattern and the substrate entry, where the acyl chain was blocked underneath the side chain of Ile62 (Figure 9A). Although Val43 occasionally exhibited torsional changes in its side chain, these did not correlate with acyl-chain entry. Leu37 and Met44 displayed behaviors similar to holo-*Ec*ACP, with their side chains oriented outward, and both experienced significant fluctuations due to solvent exposure.

In the case of octanoyl-*Ec*ACP, the acyl-chain length efficiently occupies sub-pocket I, remaining there for extended periods. The chain approaches closer to Leu42 of the α2-helix and Ala68 of the α4-helix, enhancing the potential for hydrophobic interactions (Figure 9B). This observation is consistent with chemical-shift perturbation data, which show significant increases. Additionally, residues such as Phe28 and Glu47 underwent notable structural changes when the acyl chain exceeds eight carbons, indicating increased flexibility and interaction potential (Figure 9C). For example, Glu47 tended to rotate about −180° when the acyl-chain length reached more than 12 carbons, compared to around −70° (Figure S3B). Asp56 similarly exhibited a χ1 torsion angle around 60° in the octanoyl form. This change is thought to be due to an increase in the contact area with the residues as a result of the expansion of the loop.

Lauroyl-*Ec*ACP with a 12-carbon chain marks the point at which the acyl chain begins to invade sub-pocket II. Gating effects caused by Leu42 are evident, with the acyl chain approaching Val7, located at the bottom of sub-pocket II. Corresponding changes in NMR data support this movement for chains with 12 or more carbons (Figure 9D). To study movement dynamics from sub-pocket I to II, palmitoyl-*Ec*ACP with a 16-carbon chain was modeled, revealing that Leu42 and Leu46 act as gates. Each leucine bends in opposite directions, opening a pathway for the acyl chain to orient toward sub-pocket II and thereby approach Val7. Subsequently, the palmitoyl chain exhibits an exit pathway from the cavity, exposing the long chain to the solvent, as shown in snapshots at 300 ns, 539.2 ns, and 719.9 ns, respectively. Monitoring the side-chain torsion angle (χ1) during the simulations showed that, upon opening toward sub-pocket II, Leu42 and Leu46 adopted dihedrals of approximately −70° and −180°, respectively (Figure S3B). This gating behavior becomes prominent starting with lauroyl-*Ec*ACP, and we suggest that it is consistent with the CSP for lauroyl-*Ec*ACP in Figure 7A, in which Val7 rises above the average.

As the acyl-chain length increased, structural changes became more pronounced. Using Glu60 and Asp56 on the α3 helix as a reference, we observed substantial positional shifts associated with acyl-chain ingress and egress; concomitantly, the distance to Val40 on the α2 helix increased, indicating deeper penetration of the acyl chain (Figure 9E). Asp56 on the α3 helix exhibits high R_2_ rate, indicating outward movement of the α3 helix to accommodate longer acyl chains, as observed in MD simulation, consistence with backbone dynamics data. The α2α3 loop region, which clusters negatively charged metal-binding sites, also expanded and exhibited greater fluctuation as the chain lengthened (Figure 9F). Phe28, located near the cavity entrance, tended to tilt in a consistent direction as larger chains were accommodated, further supporting their prominent R_2_ rate observed in our backbone dynamics analyses. This expansion of helices and loops ultimately led to an increase in the radius of gyration, indicating overall structural swelling, with the α2α3 loop exhibiting notable enlargement (Figure 9G).

**Figure 10:**
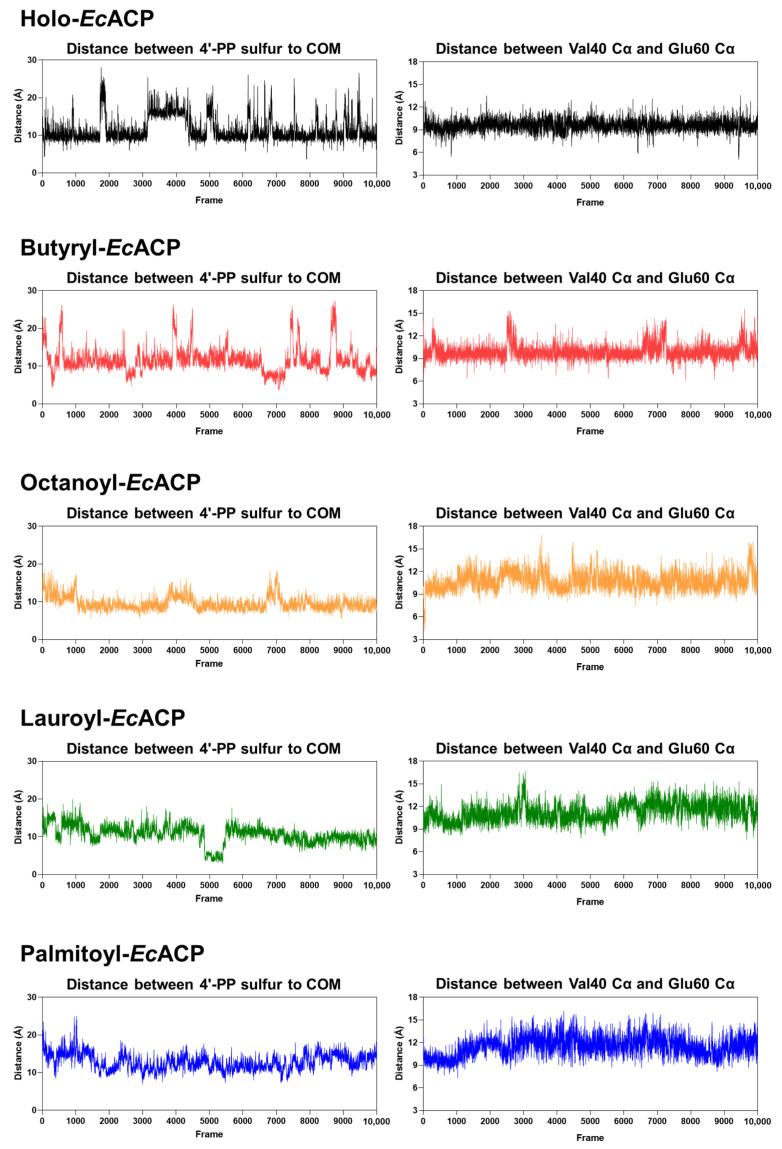
Distance analysis of *Ec*ACP extending its acyl-chain length. The left panel shows the distance between the sulfur atom on the phosphopantetheine group and the center of mass (COM), reflecting the level of acyl-chain sequestration—smaller values indicate tighter binding. The right panel displays the distance between the Cα atoms of Val40 and Glu60, representing the spatial relationship between the α2 and α3 helices.

The decrease in the distance between the center of mass (COM)—reflecting the extent of acyl-chain insertion—and the sulfur atom of the 4′-PP arm shows a tendency to correspond to the increase in the distance between the α2-helix (Val40) and the α3-helix (Glu60) (Figure 10). This trend is observed from holo- to octanoyl-*Ec*ACP but diverges starting from lauroyl-*Ec*ACP. Considering the similar pattern observed in the radius of gyration analysis (Figure 9G), it suggests that *EcACP* can optimally accommodate an acyl chain of up to eight carbons within its cavity [52].

## 3. Discussion

This study provides profound insights into the structural dynamics and stabilization mechanisms of *Ec*ACP, which is the most extensively studied bacterial ACP and a critical player in fatty acid synthesis. Combining NMR spectroscopy and MD simulations, we elucidate the crucial role of divalent metal ions, particularly Ca^2+^, in stabilizing *Ec*ACP, while also revealing how its conformational landscape adapts to varying acyl-chain lengths. This is the first to present an integration study of R_1_, R_2_, and hNOE backbone relaxation data with MD simulations on various acylate forms of *Ec*ACP, demonstrating that ACP dynamics are precisely regulated by acyl-chain length, closely associated with chain-flipping mechanisms governed by the movement of the α3-helix. These findings significantly advance our understanding of ACP’s functional plasticity and open new ways of targeting fatty acid synthesis in bacterial pathogens.

Our comprehensive investigation revealed the critical role of Ca^2+^ in stabilizing *Ec*ACP, and, notably, we report the first high-resolution solution structure of *Ec*ACP determined in the presence of Ca^2+^—an essential ion for fatty acid synthesis. This structural elucidation may provide fundamental insights into how calcium ions regulate *Ec*ACP’s stability and function within the context of FAS. NMR spectroscopy offered detailed insights into two Ca^2+^ binding sites, showing specific CSPs indicative of direct coordination and local conformational stabilization. The solution structure in the presence of Ca^2+^ further revealed a notable inward conformation of metal-binding site A, with Phe28 deeply embedded within the hydrophobic cavity, likely stabilized through Ca^2+^-mediated interactions.

**Figure 11:**
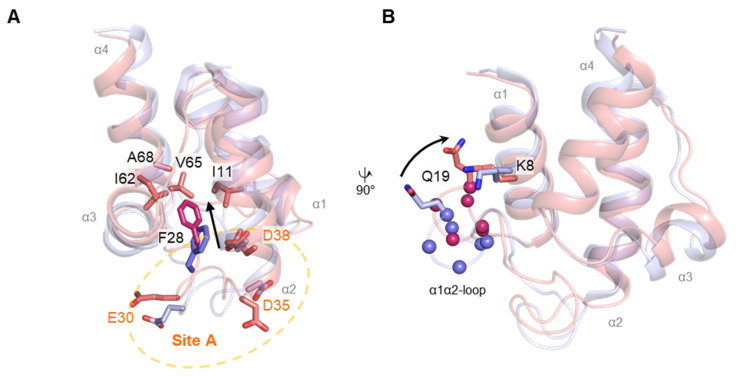
Structural comparison between holo-*Ec*ACP solution structures under different metal conditions. Holo-*Ec*ACP structures in the presence of Ca^2+^ (PDB ID 9WAB, this study) and obtained with K^+^ ion (PDB ID 2K93) are shown in pink and purple, respectively [16]. (**A**) Comparison of positioning of Phe28. Phe28 (magenta) is oriented deeper into the cavity (indicated by a black arrow), forming hydrophobic contacts with Ile11, Ile62, Val65, and Ala68. Metal-binding site A is highlighted with a yellow dashed circle. (**B**) Residues Val17–Val22 within the α1α2-loop, shown as spheres, shift toward the α1-helix, positioning Gln19 closer to Lys8 (indicated by a black arrow).

We compared our NMR structure of *Ec*ACP with Ca^2+^ to a previously reported holo-*Ec*ACP solution structure (PDB ID: 2K93) determined in potassium phosphate buffer. In the Ca^2+^-bound structure, coordination at metal-binding site A appears to shift Phe28 more inward into the hydrophobic cavity, enhancing interactions with surrounding residues such as Ile12, Ile62, Val65, and Ala68, leading to a more tightly packed cavity (Figure 11A). Notably, Gln19 near metal-binding site A exhibits a high hNOE value within the α1α2-loop, indicating enhanced local stability. In the Ca^2+^-bound structure, Gln19 (Val17–Val22) is positioned closer to Lys8 compared to the structure with K^+^ monovalent ion, and a hydrogen bond between Gln19 and Lys8 was observed in MD simulations, suggesting a metal-induced structural rearrangement.

Complementing these findings, CD experiments demonstrated a remarkable increase in structural stability upon calcium addition, with melting temperatures rising by more than 10 °C. The DSC data further supported this, revealing a higher T_m_ and a substantial increase in calorimetric enthalpy (16.7 kcal mol^−1^ increase in ΔH_cal_), indicating a more thermodynamically stable folded state facilitated by Ca^2+^ binding compared to K^+^ binding. These observations are consistent with the observed enhanced thermal stability under Ca^2+^ conditions compared to K^+^, as evidenced by the increased T_m_ values from both DSC and CD analyses.

Through structural and dynamics analysis, these findings highlight that Ca^2+^ not only mitigates electrostatic repulsion—given the highly negatively charged surface of *Ec*ACP—but also prompts specific conformational changes that reinforce the hydrophobic core and stabilize critical structural regions, such as the α1α2-loop and α3-helix. Notably, Gln19, situated near metal-binding site A, displayed a high hNOE value within the α1α2-loop, further corroborated by MD simulations, which suggest that Lys8 forms a hydrogen bond with Gln19 in the Ca^2+^-bound structure, contributing to enhanced local stability.

The Ca^2+^ titration experiments monitored by NMR CSPs confirmed binding at known metal-binding sites, with the most significant chemical shift change observed for Ile54—located at the cavity bottom—suggesting structural rearrangement rather than direct metal coordination for Ile54 (Figure S1). Additional Mn^2+^ titration revealed conformational changes in the nearby α1α2-loop, indicating that Ca^2+^ binding not only stabilizes the coordination site but also promotes conformational tightening in adjacent flexible regions. Overall, divalent cations like Ca^2+^ may play a key role in enhancing the structural stability and functional integrity of *Ec*ACP in bacterial FAS.

In the H/D exchange experiments, residues Ile11 and Ile72 stand out as key residues critical to the global folding stability of *Ec*ACP. Their high protection factors indicate their important role in maintaining a stable hydrophobic core, which is essential for the protein’s structural integrity. Ile11, located within the α1-helix at the middle of the cavity, and Ile72, situated at the bottom of the α4-helix, occupy strategic positions that are highly conserved among bacterial ACPs (Figure 1 and Figure 3B,C). The hydrophobic packing mediated by Ile11 appears vital for stability; notably, in thermophilic ACPs, such as *Tm*ACP, the equivalent residue, Ile15, demonstrates even higher amide proton protection, with ΔG_local_ exceeding 8 kcal mol^−1^ and surpassing the global stability (ΔG_global_), highlighting its crucial contribution to structural robustness [13]. The increased stability of *Tm*ACP can partly be attributed to minimized electrostatic repulsion due to additional basic residues, which enhance hydrophobic interactions. Supporting this, mutation of Ile15 to alanine in *Tm*ACP weakens packing and reduces protection, emphasizing the importance of hydrophobic contacts—a result likely paralleled if Ile11 can be mutated in *Ec*ACP, a hypothesis warranting future validation. Additionally, Ile72, positioned at the α4-helix, forms part of the hydrophobic triad at the bottom of the cavity, contributing similarly to the highest ΔG_local_ value; its role in preserving the hydrophobic core further underscores its significance in stabilizing *Ec*ACP’s folded state.

Spin-relaxation experiments provide precise insights into *Ec*ACP backbone dynamics, and MD simulations deepen these findings by offering atomistic views of dynamic behavior. In this study, combining both approaches revealed how *Ec*ACP accommodates different acyl-chain lengths (Figure 8, Figure 9 and Figure 10) and identified key residues involved in acyl-chain binding. Notably, Phe28 and Ile54 showed high R_2_ rates specifically in the butyryl form, which MD simulations confirmed by showing that the short chain cannot be stably sequestered and frequently exchanges between sequestered and solvent-exposed states. This dynamic exchange explains the elevated R_2_ rates, demonstrating the synergy of experimental and computational approaches in elucidating *Ec*ACP’s flexibility and substrate interactions.

Furthermore, our NMR and MD analyses demonstrated that the α3-helix (Asp56–Lys61) undergoes notable conformational expansion and outward movement as the acyl chain lengthens. Increased CSPs, especially in Glu60, highlight this structural change. In the octanoyl form, low hNOE values across the α3-helix and nearby loops, along with a high R_2_ rate for Asp56, indicate greater flexibility to accommodate longer chains. These experimental results are corroborated by MD simulations, which show notable fluctuations in Asp56 and Glu60, highlighting the dynamic adjustment of the α3-helix with increasing acyl-chain size (Figure 9E). This flexibility likely plays a key role in acyl-chain transfer to partner enzymes via the chain-flipping mechanism.

These findings offer insight into the structural adjustments necessary for *Ec*ACP to reach its typical biological endpoint of synthesizing C16 or C18 fatty acids. The CSP (Figure 6) and backbone dynamics data (Figure 7) from NMR experiments of acyl-*Ec*ACP consistently demonstrated chain-length-dependent conformational changes, most notably the outward displacement of the α3-helix. This is further corroborated by MD simulations, which show conformational switching of Leu42 and Leu46 to open sub-pocket II, thereby facilitating the accommodation of longer chains. Moreover, previous studies have shown that as the acyl cavity approaches its maximum volume, incomplete sequestration of longer acyl chains leads to elevated hydrolysis rates [32,53]. This trend is consistent with our MD-based analysis, including radius of gyration (Figure 9G) and distance analysis (Figure 10). Collectively, these findings align well with the biological necessity for *Ec*ACP to accommodate long-chain fatty acids.

One limitation of our study is the challenge in obtaining reliable NMR data for longer acyl chains exceeding C12, due to rapid hydrolysis and the long acquisition times required for backbone dynamics experiments. Consequently, we relied on MD simulations to investigate their conformational behavior, focusing on the motions of Leu42 and Leu46, which serve as switch pivots in the gating mechanism for sub-pocket II (Figure 9D). These residues rotate in opposite directions to create a pathway for longer acyl chains. Additionally, fluctuations in negatively charged residues such as Glu47, Glu51, and Asp56, shown in MD simulations, suggest that electrostatic repulsion drives cavity expansion and conformational looseness, facilitating longer-chain accommodation (Figure 9F). Meanwhile, residues like Ala68 remain oriented inward to maintain hydrophobic stability. To overcome the limitations caused by rapid hydrolysis during solution NMR experiments, future studies may incorporate acyl–acyl carrier protein synthetase, which attaches fatty acids to holo-ACP in an ATP-dependent manner [54], to suppress hydrolysis and stabilize long-chain acyl-*Ec*ACP samples for further investigation. Future studies involving targeted mutational analyses and advanced experimental techniques to investigate ACP-enzyme interactions are essential for directly capturing these dynamic processes. Such efforts could significantly enhance our understanding of substrate specificity and the mechanistic functions of ACPs in their interactions with enzymes during fatty acid biosynthesis.

Taken together, this study demonstrates how divalent cation Ca^2+^ coordination stabilizes *Ec*ACP and reveals how acyl-chain length modulates its conformational dynamics. By integrating advanced NMR techniques with MD simulations, we have uncovered the fundamental mechanisms driving *Ec*ACP’s structural adaptability and functional flexibility. This combined approach has elucidated the dynamic behavior of key regions and residues, revealing how structural flexibility regulates its function in fatty acid biosynthesis. These insights significantly advance our knowledge of *Ec*ACP’s conformational adaptability, highlighting the importance of developing strategies to inhibit its dynamics, which could be crucial for effectively targeting fatty acid biosynthesis.

## 4. Materials and Methods

### 4.1. Cloning, Expression, and Purification of EcACP

The acpP gene was amplified from *E. coli* K12 genomic DNA and inserted into the multiple cloning site of the pET-21a expression vector via Nde I and Xho I restriction enzymes. Recombinant plasmids were introduced into *E. coli* BL21(DE3) competent cells and used for protein expression. To produce the isotope-labelled proteins required for NMR analysis, the cells were cultured in an M9 minimal medium that was supplemented with either 0.5 g of ^15^NH_4_Cl (Cambridge Isotope Laboratories, Andover, MA, USA) for two-dimensional spectra, or with 0.5 g of ^15^NH_4_Cl and 1.0 g of ^13^C-glucose (Cambridge Isotope Laboratories, Andover, MA, USA) for three-dimensional experiments. For other experiments, LB medium was used instead. Protein overexpression was induced via supplementation with 0.5 Mm isopropyl β-D-thiogalactopyranoside (IPTG) when the optical density value of the culture medium reached between 0.8 and 1.0. Then, the culture was incubated at 25 °C for 16 h.

Purification of *Ec*ACP was achieved through sequential FPLC steps exploiting its physicochemical properties: ion-exchange chromatography (HiTrap™ QFF and Resource™ Q, GE Healthcare Bio-Sciences, Uppsala, Sweden) and size-exclusion chromatography (Superdex 75 16/600, GE Healthcare Bio-Science, Uppsala, Sweden). The apo-protein was subsequently converted into the holo- or acylated forms by incubations with recombinant *Ec*ACPS at 25 °C for 16 h in 25 mM of Tris-HCl buffer (pH 8.0) containing 20 mM MgCl_2_.

### 4.2. Circular Dichroism Experiments

The secondary structure of *Ec*ACP under varying temperatures was analyzed using far-UV CD with a J-810 spectropolarimeter (Jasco, Tokyo, Japan). Protein samples were prepared at a final concentration of 30 μM in 25 mM of MES buffer (pH 6.1) containing 5 mM of dithiothreitol (DTT). Four conditions were tested: buffer supplemented with 5 mM CaCl_2,_ 5 mM MgCl_2_, 5 mM KCl, and a sample without added metal ions. The 1 mm path-length quartz cuvette was used for measurements. CD spectra were acquired from 200 to 250 nm, with a data pitch of 0.1 nm. The sample temperature was gradually raised from 25 to 100 °C, and thermal unfolding was tracked by monitoring changes in mean residue ellipticity (θ) at 222 nm, which reflects α-helical content.

### 4.3. Differential Scanning Calorimetry

Thermal properties were analyzed using DSC experiments on a MicroCal PEAQ-DSC system (Malvern Instruments, Malvern, UK) at the Korea Basic Science Institute (KBSI) (Ochang, Cheongju, Republic of Korea). Then, 5 mg/mL of protein samples was prepared in 50 mM sodium acetate buffer (pH 5.6) with 5 mM CaCl_2_ or 5 mM MgCl_2_. The DSC thermograms were collected over a temperature range of 20–100 °C, at a constant rate 1 °C/min. Data acquisition and baseline subtraction were carried out with PEAQ-DSC measurement software (v1.64), and thermodynamic parameters, including the T_m_, ΔH_cal_, and C_p_, were extracted by nonlinear least-squares fitting, assuming a two-state transition model.

### 4.4. NMR Experiments and Assignment

All NMR experiments in this study were conducted on Bruker Avance 700 MHz and 900 MHz spectrometers at the KBSI (Ochang, Cheongju, Republic of Korea). *Ec*ACP samples, either single- or double-isotope-labeled, were prepared at concentrations of 0.4–0.5 mM in a buffer consisting of 25 mM MES (pH 6.1), supplemented with 5 mM CaCl_2_ and 5 mM DTT, in a 9:1 (*v*/*v*) of H_2_O/D_2_O mixture. 2,2-Dimethyl-2-silapentane-5-sulfonate (DSS) served as the internal chemical shift reference, and 0.02% NaN_3_ was added for the purpose of preventing microbial growth. All spectra were processed with NMRPipe [55] and analyzed using NMRFAM-Sparky [56].

Backbone resonance assignments were obtained from HNCO, HNCACB, and CBCA(CO)NH experiments, while side-chain resonances were assigned using CC(CO)NH, HBHA(CO)NH, H(CCO)NH, and HCCH-TOCSY. Assignments were further validated by ^1^H-^15^N-^1^H NOESY-HSQC and ^1^H-^13^C-^1^H NOESY-HSQC spectra.

The RDCs of the backbone amide N-H bonds were determined by comparing the isotropic and anisotropic conditions in the IPAP-HSQC spectra. For the anisotropic sample, *Ec*ACP was dissolved in a radially compressed polyacrylamide gel, enabling partial alignment [57,58,59].

### 4.5. Solution Structure Calculation

NOE cross-peaks were assigned using NMRFAM-Sparky [56]. The three-dimensional structure of holo-*Ec*ACP was calculated using the PONDEROSA-C/S package with Xplor-NIH-based calculations [60]. From the ensemble generated, the twenty structures with the lowest energy levels were selected. Constrain violations for bond angles and interproton distances within these 20 models were further checked and refined using PONDEROSA-Analyzer [61]. The structural ensemble was validated with Protein Structure Validation Software [62]. All protein structure images were prepared using PyMOL (version 3.1.3.1) [63]. The final coordinates and NOE constraints were submitted to the Protein Data Bank (PDB) (PDB ID 9WAB).

### 4.6. Hydrogen/Deuterium Exchange Experiment

For the H/D exchange analysis, a ^15^N-labeled *Ec*ACP sample was prepared at a concentration of 0.5 mM in 25 mM of MES buffer (pH 6.1) containing 5 mM DTT and 0.02% NaN_3_ with 5 mM CaCl_2_. A set of ^1^H-^15^N HSQC spectra was acquired every 10 min at 25 °C for a total period of 1000 min. The decay of amide proton signals over time was quantified from the decrease in peak intensities. The exchange rate constants (k_ex_) were obtained by fitting the intensity decay to a single-exponential function:

$$I=I_{0}\exp\left({-k}_{ex}t\right)+C$$

where I represents the NH resonance intensity at time t, and I_0_ is the initial intensity. The experimentally determined exchange rate constant, k_prot_, was treated as equivalent to k_ex_; the protection factor (P) was calculated as the ratio of the k_rc_ to k_prot_; and ΔG_local_ was subsequently determined using the following relationship [64]:

$${\Delta G}_{local}=-RT\;ln(\frac{k_{prot}}{k_{rc}})$$

### 4.7. Chemical Denaturation Experiments

The global unfolding of *Ec*ACP under chemical denaturant was evaluated by far-UV CD experiment. Protein samples (30 μM) were prepared in 25 mM of MES buffer (pH 6.1) containing 5 mM of DTT, either with or without 5 mM CaCl_2_. Various Gdn-HCl concentrations were added to the protein samples, followed by 25 °C for 12 h to ensure that equilibrium was reached. CD spectra were recorded over the wavelength range of 200–250 nm, in steps of 0.1 nm. Ther denaturation curves were analyzed, assuming a reversible two-state equilibrium between the native (N) and unfolded (U) states. Data fitting was performed using the linear extrapolation model [65,66,67,68]. The global free energy of unfolding (ΔG_global_) was calculated following the procedure in a previous study [13].

### 4.8. Metal Titration Experiments

Successive ^1^H-^15^N HSQC spectra were acquired following the stepwise addition of Ca^2+^ and Mn^2+^ to final concentrations of 0 mM, 0.5 mM, 1 mM, 2 mM, 5 mM, 10 mM, 20 mM, 30 mM for Ca^2+^; and 0 mM, 0.001 mM, 0.005 mM, 0.01 mM, 0.02 mM, and 0.05 mM, 0.1 mM for Mn^2+^ to 0.3 mM of *Ec*ACP, respectively. For the Ca^2+^ titration, CSP analysis was carried out to identify residues exhibiting significant chemical shift changes. In the Mn^2+^ titration, peak intensities at each titration data point were normalized against the initial spectrum to monitor residue-specific decrease in signal intensity.

### 4.9. Chemical Shift Perturbations

CSP analysis was also performed to assess the effect of acylation on the structural properties of *Ec*ACP. ^1^H-^15^N HSQC spectra for 0.5 mM of apo-, holo-, butyryl-, octanoyl-, lauroyl-*Ec*ACP were obtained from NMR experiments. The CSPs between holo-*Ec*ACP and apo- or acylated forms were calculated using the following formula:

$$∆\delta =\sqrt{0.5(∆\delta {\left(1H\right)}^{2})+(\alpha ∆\delta {\left(15N\right)}^{2}}$$

α = 0.2 for most residues, and 0.14 for glycine

### 4.10. Backbone Dynamics

Backbone dynamics of holo-, butyryl-, octanoyl-*Ec*ACP (0.5 mM) were characterized using NMR spin-relaxation measurements at 25 °C with a Bruker Avance 700 MHz spectrometer. A series of ^1^H–^15^N HSQC spectra of R_1_, R_2_, and hNOE were acquired. For R_1_ experiments, the applied relaxation delays were 0.01 s, 0.01 s, 0.05 s, 0.1 s, 0.2 s, 0.3 s, 0.3 s, 0.5 s, 0.8 s, and 1.2 s. For R_2_ experiments, delays of 0 s, 0 s, 0.01696 s, 0.03392 s, 0.05088 s, 0.05088 s, 0.0848 s, 0.013568 s, 0.022048 s, and 0.32224 s were used. The recycle delays were 2.3 and 2.0 s for R_1_ and R_2_ experiments, respectively. The hNOE experiments were performed with a recycle delay of 4.0 s and proton saturation of 3.0 s. Relaxation rates were obtained by fitting the exponential decay of peak intensities across the respective delay series.

### 4.11. Molecular Dynamics Simulations

All MD simulations were performed from the NMR structure of *Ec*ACP determined in this study (PDB ID: 9WAB). Acyl chains were covalently attached to the serine in the DSL motif using CHAARMM-GUI’s Ligand Reader & Modeler [69], with parameters generated by CGenFF (force field 3.0) [70,71,72]. Systems were prepared in CHARMM-GUI’s Solution Builder, employing a cubic water box size that maintained a 10.0 Å edge distance from the protein [73,74,75]. K^+^ and Cl^−^ were added to reach 0.15 M, neutralizing all models with TIP3P water. Simulations were performed in OpenMM 8.0.0 with the CHARMM36 m force field [76,77], ensuring consistent treatment of water models and nonbonded settings in our study. Long-range electrostatics were treated with particle-mesh Ewald (PME) under periodic boundary conditions, using a 1.2 nm real-space cutoff and an Ewald error tolerance of 5 × 10^−4^; and short-range Coulomb interactions were computed directly within the cutoff, and reciprocal-space contributions were evaluated on a mesh. Lennard–Jones interactions employed a force-switch between 1.0 and 1.2 nm, going smoothly to zero at 1.2 nm. During equilibration and production, we simulated in the NVT and NPT ensemble, respectively, with temperature held at 310.15 K, using a Langevin thermostat (friction coefficient 1 ps^−1^), as implemented in OpenMM 8.0.0. Pressure control (1 bar, isotropic Monte Carlo barostat) was applied during production only after equilibration had converged. For both equilibration and production, the lengths of all bonds that involve a hydrogen atom are constrained to use a larger integration time step. With HBonds constraints, the time step increased to about 2 fs in production and 1 fs in equilibration for Langevin dynamics. Each system underwent energy minimization for 0.125 ns, followed by the standard six-step CHARMM-GUI equilibration protocol with a 1 fs timestep. Production runs were then carried out for 1000 ns under constant temperature (310.15 K) and pressure (1 bar, isotropic), using Langevin dynamics. Production trajectories were collected for 1000 ns per replica, with three independent replicas per system. Trajectories and snapshots were analyzed and visualized using VMD 1.9.3 [78].

## 5. Conclusions

This study highlights how Ca^2+^ stabilizes *Ec*ACP and how acyl-chain length influences its structural flexibility and dynamics. CD, DSC, and H/D exchange experiments revealed that Ca^2+^ enhances the stability of *Ec*ACP, and hydrophobic interactions mediated by Ile11 and Ile72 are crucial for maintaining *Ec*ACP folding. By combining NMR and MD approaches, we gained detailed insights into how chain-length-dependent conformational changes—particularly the motion of the α3-helix acting as a molecular gatekeeper—facilitate substrate accommodation and transfer. These findings deepen our understanding of *Ec*ACP’s mechanistic flexibility and identify potential targets for designing antibacterial agents that disrupt FAS.

## Figures and Tables

**Table 1:** Comparison of the number of charged residues, isoelectric points and melting temperature among mesophilic, thermophilic, and psychrophilic ACPs.

	*Ec*ACP	*Ab*ACP ^a^	*Vh*ACP ^b^	*Br*ACP ^c^	*Tm*ACP ^d^	*Ta*ACP ^e^	*Cp*ACP ^f^
Acidic	19	19	22	20	21	21	18
Basic	5	6	5	6	9	10	2
pI ^g^	3.98	3.92	3.79	3.97	4.13	4.29	3.70
T_m_ (°C) ^h^	67.4	68.0	66.4	60.8	100.4	-	-

^a^ *Acinetobacter baumannii* ACP; ^b^ *Vibrio harveyi* ACP; ^c^ *Brucella melitensis* ACP; ^d^ *Thermotoga maritima* ACP; ^e^ *Thermus aquaticus* ACP; ^f^ *Colwellia psychrerythraea* ACP. ^g^ The isoelectric points (pI) of ACPs were calculated using the ProtParam tool [43]. ^h^ Melting temperatures (T_m_) of *Ec*ACP, *Ab*ACP, *Vh*ACP, and *Tm*ACP were determined in the presence of Ca^2+^, while the information regarding metal addition is not reported for *Br*ACP [10,13,14,44]. A dash indicates that the T_m_ value was not reported.

## Data Availability

The data presented in this study are available upon request from the corresponding author.

## Supplementary Materials

The following supporting information can be downloaded at https://www.mdpi.com/article/10.3390/ijms26189005/s1.

## Abbreviations

The following abbreviations are used in this manuscript:

ACP	Acyl carrier protein
FAS	Fatty acid synthesis
MD	Molecular dynamics
*Ec*	*Escherichia coli*
ACPS	Holo-ACP synthase
4′-PP	4′-phosphopantetheine
MDR	Multi-drug-resistant
Gdn-HCl	Guanidine hydrochloride
RDC	Residual dipolar coupling
H/D	Hydrogen–deuterium
DSC	Differential scanning calorimetry
pI	Isoelectric point
*Tm*	*Thermotoga maritima*
*Cp*	*Colwellia psychrerythraea*
*Ta*	*Thermus aquaticus*
*Ab*	*Acinetobacter baumannii*
*Vh*	*Vibrio harveyi*
T_m_	Melting temperature
CD	Circular dichroism
C_p_	Specific heat capacity
ΔH_cal_	Calorimetric enthalpy
RMSD	Root-mean-square deviation
LogP	Protection factor
k_ex_	Exchange rate
k_prot_	H/D exchange rate
k_rc_	Random coil exchange rate
ΔG_local_	Local unfolding free energy
ΔG_global_	Global unfolding free energy
[Gdn-HCl]_1/2_	Half-denaturation concentration of Gdn-HCl
CSP	Chemical shift perturbation
HSQC	Heteronuclear single quantum coherence
R_1_	Longitudinal spin relaxation
R_2_	Transverse spin relaxation
hNOE	Heteronuclear NOE
χ1	Side-chain torsion angle
COM	Center of mass
IPTG	Isopropyl β-D-thiogalactopyranoside
DTT	Dithiothreitol
DSS	2,2-Dimethyl-2-silapentane-5-sulfonate
PDB	Protein Data Bank
KBSI	Korea Basic Science Institute

## References

[1] Chan D.I., Vogel H.J. (2010). Current understanding of fatty acid biosynthesis and the acyl carrier protein. Biochem. J..

[2] Shen B., Summers R.G., Gramajo H., Bibb M.J., Hutchinson C.R. (1992). Purification and characterization of the acyl carrier protein of the Streptomyces glaucescens tetracenomycin C polyketide synthase. J. Bacteriol..

[3] Masoudi A., Raetz C.R., Zhou P., Pemble C.W.T. (2014). Chasing acyl carrier protein through a catalytic cycle of lipid A production. Nature.

[4] Wakil S.J., Stoops J.K., Joshi V.C. (1983). Fatty acid synthesis and its regulation. Annu. Rev. Biochem..

[5] Jenni S., Leibundgut M., Boehringer D., Frick C., Mikolasek B., Ban N. (2007). Structure of fungal fatty acid synthase and implications for iterative substrate shuttling. Science.

[6] Smith S., Tsai S.C. (2007). The type I fatty acid and polyketide synthases: A tale of two megasynthases. Nat. Prod. Rep..

[7] White S.W., Zheng J., Zhang Y.M., Rock C.O. (2005). The structural biology of type II fatty acid biosynthesis. Annu. Rev. Biochem..

[8] Lu Y.J., Zhang Y.M., Rock C.O. (2004). Product diversity and regulation of type II fatty acid synthases. Biochem. Cell Biol..

[9] Park J., Lee Y., Cheon D., Kim Y. (2019). Structure and dynamics of human and bacterial acyl carrier proteins and their interactions with fatty acid synthesis proteins. Biochem. Biophys. Res. Commun..

[10] Choi S., Park J., Yeon J., Jang A., Lee W.C., Kim Y. (2021). Deciphering the Binding Interactions between *Acinetobacter baumannii* ACP and β-ketoacyl ACP Synthase III to Improve Antibiotic Targeting Using NMR Spectroscopy. Int. J. Mol. Sci..

[11] Son M., Oh S., Oh Y., Cheon D., Jang A., Kim E., Kim N.K., Kim Y. (2024). Structural and dynamic insights into acyl carrier protein in *Cutibacterium acnes* reveal mechanisms for fatty acid synthesis and transport. Biochem. Biophys. Res. Commun..

[12] Park Y.G., Jung M.C., Song H., Jeong K.W., Bang E., Hwang G.S., Kim Y. (2016). Novel Structural Components Contribute to the High Thermal Stability of Acyl Carrier Protein from *Enterococcus faecalis*. J. Biol. Chem..

[13] Lee Y., Jang A., Jeong M.C., Park N., Park J., Lee W.C., Cheong C., Kim Y. (2020). Structural Characterization of an ACP from *Thermotoga maritima*: Insights into Hyperthermal Adaptation. Int. J. Mol. Sci..

[14] Chan D.I., Chu B.C., Lau C.K., Hunter H.N., Byers D.M., Vogel H.J. (2010). NMR solution structure and biophysical characterization of Vibrio harveyi acyl carrier protein A75H: Effects of divalent metal ions. J. Biol. Chem..

[15] Kim Y., Prestegard J.H. (1990). Refinement of the NMR structures for acyl carrier protein with scalar coupling data. Proteins.

[16] Wu B.N., Zhang Y.M., Rock C.O., Zheng J.J. (2009). Structural modification of acyl carrier protein by butyryl group. Protein Sci..

[17] Roujeinikova A., Simon W.J., Gilroy J., Rice D.W., Rafferty J.B., Slabas A.R. (2007). Structural studies of fatty acyl-(acyl carrier protein) thioesters reveal a hydrophobic binding cavity that can expand to fit longer substrates. J. Mol. Biol..

[18] Sharma A.K., Sharma S.K., Surolia A., Surolia N., Sarma S.P. (2006). Solution structures of conformationally equilibrium forms of holo-acyl carrier protein (PfACP) from Plasmodium falciparum provides insight into the mechanism of activation of ACPs. Biochemistry.

[19] Lee J.Y., Jeong K.W., Lee J.U., Kang D.I., Kim Y. (2009). Novel *E. coli* β-ketoacyl-acyl carrier protein synthase III inhibitors as targeted antibiotics. Bioorg. Med. Chem..

[20] Jeong K.W., Lee J., Kang D.I., Lee J.U., Shin S.Y., Kim Y. (2009). Screening of Flavonoids as Candidate Antibiotics against *Enterococcus faecalis*. J. Nat. Prod..

[21] Lee J.Y., Jeong K.W., Shin S., Lee J.U., Kim Y. (2012). Discovery of novel selective inhibitors of *Staphylococcus aureus* β-ketoacyl acyl carrier protein synthase III. Eur. J. Med. Chem..

[22] Lee J.Y., Jeong M.C., Jeon D., Lee Y., Lee W.C., Kim Y. (2017). Structure-activity relationship-based screening of antibiotics against Gram-negative *Acinetobacter baumannii*. Bioorg. Med. Chem..

[23] Lee W.C., Park J., Balasubramanian P.K., Kim Y. (2018). Elucidation of the crystal structure of FabD from the multidrug-resistant bacterium *Acinetobacter baumannii*. Biochem. Biophys. Res. Commun..

[24] Cheon D., Lee W.C., Lee Y., Lee J.Y., Kim Y. (2019). Structural basis of branched-chain fatty acid synthesis by Propionibacterium acnes β-ketoacyl acyl Carrier protein synthase. Biochem. Biophys. Res. Commun..

[25] Ha Y., Jang M., Lee S., Lee J.Y., Lee W.C., Bae S., Kang J., Han M., Kim Y. (2020). Identification of inhibitor binding hotspots in *Acinetobacter baumannii* β-ketoacyl acyl carrier protein synthase III using molecular dynamics simulation. J. Mol. Graph. Model..

[26] Mindrebo J.T., Chen A., Kim W.E., Re R.N., Davis T.D., Noel J.P., Burkart M.D. (2021). Structure and Mechanistic Analyses of the Gating Mechanism of Elongating Ketosynthases. Acs Catal..

[27] Misson L.E., Mindrebo J.T., Davis T.D., Patel A., McCammon J.A., Noel J.P., Burkart M.D. (2020). Interfacial plasticity facilitates high reaction rate of E. coli FAS malonyl-CoA:ACP transacylase, FabD. Proc. Natl. Acad. Sci. USA.

[28] Mindrebo J.T., Patel A., Kim W.E., Davis T.D., Chen A., Bartholow T.G., La Clair J.J., McCammon J.A., Noel J.P., Burkart M.D. (2020). Gating mechanism of elongating beta-ketoacyl-ACP synthases. Nat. Commun..

[29] Cronan J.E. (2014). The chain-flipping mechanism of ACP (acyl carrier protein)-dependent enzymes appears universal. Biochem. J..

[30] Beld J., Cang H., Burkart M.D. (2014). Visualizing the Chain-Flipping Mechanism in Fatty-Acid Biosynthesis. Angew. Chem. Int. Ed..

[31] Arya R., Sharma B., Dhembla C., Pal R.K., Patel A.K., Sundd M., Ghosh B., Makde R.D., Kundu S. (2019). A conformational switch from a closed apo- to an open holo-form equips the acyl carrier protein for acyl chain accommodation. Biochim. Biophys. Acta Proteins Proteom..

[32] Sztain T., Bartholow T.G., Lee D.J., Casalino L., Mitchell A., Young M.A., Wang J., McCammon J.A., Burkart M.D. (2021). Decoding allosteric regulation by the acyl carrier protein. Proc. Natl. Acad. Sci. USA.

[33] Colizzi F., Masetti M., Recanatini M., Cavalli A. (2016). Atomic-Level Characterization of the Chain-Flipping Mechanism in Fatty-Acids Biosynthesis. J. Phys. Chem. Lett..

[34] Colizzi F., Recanatini M., Cavalli A. (2008). Mechanical Features of *Plasmodium falciparum* Acyl Carrier Protein in the Delivery of Substrates. J. Chem. Inf. Model..

[35] Frederick A.F., Kay L.E., Prestegard J.H. (1988). Location of divalent ion sites in acyl carrier protein using relaxation perturbed 2D NMR. FEBS Lett..

[36] Gong H., Murphy A., McMaster C.R., Byers D.M. (2007). Neutralization of acidic residues in helix II stabilizes the folded conformation of acyl carrier protein and variably alters its function with different enzymes. J. Biol. Chem..

[37] Horvath L.A., Sturtevant J.M., Prestegard J.H. (1994). Kinetics and thermodynamics of thermal denaturation in acyl carrier protein. Protein Sci..

[38] Oh S., Lee C., Son M., Yeon J., Kim Y. (2024). Dynamics of acyl carrier protein in de novo fatty acid synthesis by *Enterococcus faecalis* based on NMR spectroscopy and molecular dynamics simulation. J. Anal. Sci. Technol..

[39] Beld J., Sonnenschein E.C., Vickery C.R., Noel J.P., Burkart M.D. (2014). The phosphopantetheinyl transferases: Catalysis of a post-translational modification crucial for life. Nat. Prod. Rep..

[40] Stenstroem O., Champion C., Lehner M., Bouvignies G., Riniker S., Ferrage F. (2022). How does it really move? Recent progress in the investigation of protein nanosecond dynamics by NMR and simulation. Curr. Opin. Struct. Biol..

[41] Kümmerer F., Orioli S., Harding-Larsen D., Hoffmann F., Gavrilov Y., Teilum K., Lindorff-Larsen K. (2021). Fitting Side-Chain NMR Relaxation Data Using Molecular Simulations. J. Chem. Theory Comput..

[42] Champion C., Lehner M., Smith A.A., Ferrage F., Bolik-Coulon N., Riniker S. (2024). Unraveling motion in proteins by combining NMR relaxometry and molecular dynamics simulations: A case study on ubiquitin. J. Chem. Phys..

[43] Wilkins M.R., Gasteiger E., Bairoch A., Sanchez J.C., Williams K.L., Appel R.D., Hochstrasser D.F. (1999). Protein identification and analysis tools in the ExPASy server. Methods Mol. Biol..

[44] Barnwal R.P., Kaur M., Heckert A., Gartia J., Varani G. (2020). Comparative structure, dynamics and evolution of acyl-carrier proteins from Borrelia burgdorferi, Brucella melitensis and Rickettsia prowazekii. Biochem. J..

[45] Paul S., Ishida H., Nguyen L.T., Liu Z., Vogel H.J. (2017). Structural and dynamic characterization of a freestanding acyl carrier protein involved in the biosynthesis of cyclic lipopeptide antibiotics. Protein Sci..

[46] Rivas M., Courouble V.C., Baker M.C., Cookmeyer D.L., Fiore K.E., Frost A.J., Godbe K.N., Jordan M.R., Krasnow E.N., Mollo A. (2018). The Effect of Divalent Cations on the Thermostability of Type II Polyketide Synthase Acyl Carrier Proteins. AIChE J..

[47] Chan D.I., Stockner T., Tieleman D.P., Vogel H.J. (2008). Molecular Dynamics Simulations of the Apo-, Holo-, and Acyl-forms of *Escherichia coli* Acyl Carrier Protein. J. Biol. Chem..

[48] Andrec M., Hill R.B., Prestegard J.H. (1995). Amide exchange rates in Escherichia coli acyl carrier protein: Correlation with protein structure and dynamics. Protein Sci..

[49] Xie Y.Z., Liu Z.Y., Qin H.W., Zhao H., Zhang W.Q., Xiao C.L., Wang X., Yang X.M., Wang F.J. (2025). Ultraviolet Photodissociation Mass Spectrometry Captures the Acyl Chain Length-Dependent Conformation Dynamics of Acyl Carrier Protein. J. Am. Chem. Soc..

[50] Mayo K.H., Prestegard J.H. (1985). Acyl Carrier Protein from *Escherichia Coli*—Structural Characterization of Short-Chain Acylated Acyl Carrier Proteins by NMR. Biochemistry.

[51] Roujeinikova A., Baldock C., Simon W.J., Gilroy J., Baker P.J., Stuitje A.R., Rice D.W., Slabas A.R., Rafferty J.B. (2002). X-ray crystallographic studies on butyryl-ACP reveal flexibility of the structure around a putative acyl chain binding site. Structure.

[52] Cronan J.E. (1982). Molecular properties of short chain acyl thioesters of acyl carrier protein. J. Biol. Chem..

[53] Zornetzer G.A., Tanem J., Fox B.G., Markley J.L. (2010). The length of the bound fatty acid influences the dynamics of the acyl carrier protein and the stability of the thioester bond. Biochemistry.

[54] Beld J., Finzel K., Burkart M.D. (2014). Versatility of acyl-acyl carrier protein synthetases. Chem. Biol..

[55] Delaglio F., Grzesiek S., Vuister G.W., Zhu G., Pfeifer J., Bax A. (1995). NMRPipe: A multidimensional spectral processing system based on UNIX pipes. J. Biomol. NMR.

[56] Lee W., Tonelli M., Markley J.L. (2015). NMRFAM-SPARKY: Enhanced software for biomolecular NMR spectroscopy. Bioinformatics.

[57] Chou J.J., Gaemers S., Howder B., Louis J.M., Bax A. (2001). A simple apparatus for generating stretched polyacrylamide gels, yielding uniform alignment of proteins and detergent micelles. J. Biomol. NMR.

[58] Sass H.J., Musco G., Stahl S.J., Wingfield P.T., Grzesiek S. (2000). Solution NMR of proteins within polyacrylamide gels: Diffusional properties and residual alignment by mechanical stress or embedding of oriented purple membranes. J. Biomol. NMR.

[59] Cordier F., Dingley A.J., Grzesiek S. (1999). A doublet-separated sensitivity-enhanced HSQC for the determination of scalar and dipolar one-bond J-couplings. J. Biomol. NMR.

[60] Lee W., Stark J.L., Markley J.L. (2014). PONDEROSA-C/S: Client-server based software package for automated protein 3D structure determination. J. Biomol. NMR.

[61] Lee W., Cornilescu G., Dashti H., Eghbalnia H.R., Tonelli M., Westler W.M., Butcher S.E., Henzler-Wildman K.A., Markley J.L. (2016). Integrative NMR for biomolecular research. J. Biomol. NMR.

[62] Bhattacharya A., Tejero R., Montelione G.T. (2007). Evaluating protein structures determined by structural genomics consortia. Proteins.

[63] Schrodinger, LLC (2015). The PyMOL Molecular Graphics System, version 1.8.

[64] Bai Y., Milne J.S., Mayne L., Englander S.W. (1993). Primary structure effects on peptide group hydrogen exchange. Proteins.

[65] Santoro M.M., Bolen D.W. (1992). A test of the linear extrapolation of unfolding free energy changes over an extended denaturant concentration range. Biochemistry.

[66] Pace C.N. (1986). Determination and analysis of urea and guanidine hydrochloride denaturation curves. Methods Enzymol..

[67] Schellman J.A. (1987). The thermodynamic stability of proteins. Annu. Rev. Biophys. Biophys. Chem..

[68] Padmanabhan S., Laurents D.V., Fernandez A.M., Elias-Arnanz M., Ruiz-Sanz J., Mateo P.L., Rico M., Filimonov V.V. (1999). Thermodynamic analysis of the structural stability of phage 434 Cro protein. Biochemistry.

[69] Kim S., Lee J., Jo S., Brooks C.L., Lee H.S., Im W. (2017). CHARMM-GUI ligand reader and modeler for CHARMM force field generation of small molecules. J. Comput. Chem..

[70] Vanommeslaeghe K., Hatcher E., Acharya C., Kundu S., Zhong S., Shim J., Darian E., Guvench O., Lopes P., Vorobyov I. (2010). CHARMM general force field: A force field for drug-like molecules compatible with the CHARMM all-atom additive biological force fields. J. Comput. Chem..

[71] Vanommeslaeghe K., MacKerell A.D. (2012). Automation of the CHARMM General Force Field (CGenFF) I: Bond perception and atom typing. J. Chem. Inf. Model..

[72] Vanommeslaeghe K., Raman E.P., MacKerell A.D. (2012). Automation of the CHARMM General Force Field (CGenFF) II: Assignment of bonded parameters and partial atomic charges. J. Chem. Inf. Model..

[73] Brooks B.R., Brooks C.L., Mackerell A.D., Nilsson L., Petrella R.J., Roux B., Won Y., Archontis G., Bartels C., Boresch S. (2009). CHARMM: The biomolecular simulation program. J. Comput. Chem..

[74] Jo S., Kim T., Iyer V.G., Im W. (2008). CHARMM-GUI: A web-based graphical user interface for CHARMM. J. Comput. Chem..

[75] Lee J., Cheng X., Swails J.M., Yeom M.S., Eastman P.K., Lemkul J.A., Wei S., Buckner J., Jeong J.C., Qi Y. (2016). CHARMM-GUI Input Generator for NAMD, GROMACS, AMBER, OpenMM, and CHARMM/OpenMM Simulations Using the CHARMM36 Additive Force Field. J. Chem. Theory Comput..

[76] Eastman P., Galvelis R., Pelaez R.P., Abreu C.R.A., Farr S.E., Gallicchio E., Gorenko A., Henry M.M., Hu F., Huang J. (2023). OpenMM 8: Molecular Dynamics Simulation with Machine Learning Potentials. arXiv.

[77] Huang J., Rauscher S., Nawrocki G., Ran T., Feig M., de Groot B.L., Grubmuller H., MacKerell A.D. (2017). CHARMM36m: An improved force field for folded and intrinsically disordered proteins. Nat. Methods.

[78] Humphrey W., Dalke A., Schulten K. (1996). VMD: Visual molecular dynamics. J. Mol. Graph..

