# Supplementary material for: Structural and Dynamic Insights into Acyl Carrier Protein upon Metal Binding and Acylation Revealed by NMR Spectroscopy and MD Simulations
Source: Int J Mol Sci. 2025 Sep 16;26(18):9005. doi: 10.3390/ijms26189005 (PMC12469350; doi:10.3390/ijms26189005)
Supplement: Supplementary file 1 [file ijms-26-09005-s001.zip › ijms-3857299-supplementary.pdf]

Supplementary Information

Structural and Dynamic Insights into Acyl Carrier Protein upon Metal binding and Acylation Revealed by NMR spectroscopy and MD simulations

Chae Yeong Lee[‡], Sungchan Jang[‡], Hyunjoon Cho[‡], Min-Cheol Jeong, Yoojin Oh, and Yangmee Kim[*]

Department of Bioscience and Biotechnology, Konkuk University, 120 Neungdong-ro, Gwangjin-gu, Seoul 05029, Republic of Korea

*** Correspondence**

Yangmee Kim, Department of Bioscience and Biotechnology, Konkuk University, Neungdong-ro 120, Gwangjin-gu, Seoul 05029, Republic of Korea

Email: ymkim@konkuk.ac.kr

Telephone: +822-450-3421

Fax: +822-447-5987

Table of Contents

Supplementary Tables

Table S1. Statistics of the 20 lowest energy structures of holo-*Ec*ACP (PDB ID: 9WAB)

Supplementary Figures

Figure S1. NMR Ca^{2+} titration of holo-*Ec*ACP.

Figure S2. NMR Mn^{2+} titration of holo-*Ec*ACP.

Figure S3. Numerical data of *Ec*ACP.

Table S1. Statistics of the 20 lowest energy structures of holo-*Ec*ACP (PDB ID: 9WAB).

**Restraints** [a]	
Total	1416
Conformationally restricting distance constraints	
Short Range [(i-j) ≤ 1]	283
Medium Range [1 < (i-j) ≤ 5]1	555
Long Range [(i-j) ≥ 5]	298
Dihedral angle constraints	
Phi	68
Psi	69
Residual dipolar coupling constraints	75
Hydrogen bond constraints	68
Xplor-NIH pseudo-potential energy (kJ/mol) [b]	3133
**Average RMSD to the mean Xplor-NIH coordinates [Å]** [c]	
Backbone atoms (all residues / order residues) [d]	0.2/0.2
Heavy atoms (all residues / order residues) [d]	0.5/0.5
**Ramachandran plot summary from PROCHECK [%]** [c]	
Most favored regions	90.8
Additionally allowed regions	9.2
Generously allowed regions	0
Disallowed regions	0
**Average number of violations per Xplor-NIH conformer** [b]	
Distance constraint violations (> 0.2Å)	0
Angle constraint violations (> 10°)	0

[a] The solution structure of holo-*Ec*ACP was calculated by using Xplor-NIH-based calculations in PONDEROSA C/S [1]. [b] Xplor-NIH pseudo-potential energy and all violations of residues are analyzed by using PONDEROSA-Analyzer [2]. [c] The final 20 lowest energy structures were evaluated by Protein Structure Validation Software suite (PSVS) [3]. [d] Ordered residues are from T2 to Q76.

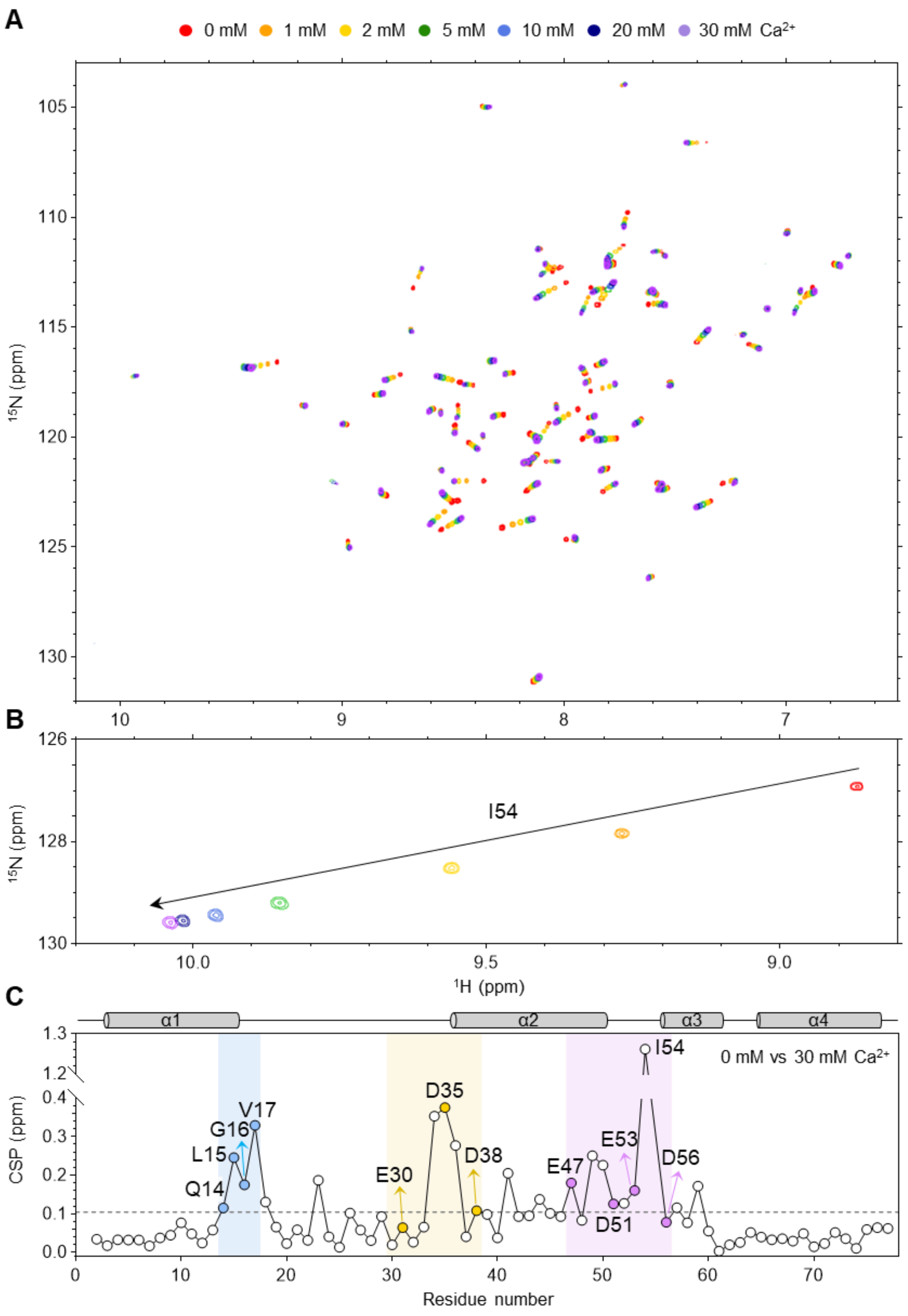

Figure S1. NMR Ca^{2+} titration of holo-*Ec*ACP. (A) Overlaid ^{1}H-^{15}N HSQC spectra of holo-*Ec*ACP at various concentrations of Ca^{2+}; 0 mM (red), 1 mM (orange), 2 mM (yellow), 5 mM (green), 10 mM (blue), 20 mM (navy), and 30 mM (purple). **(B)** Peak trace of I54 which exhibited the largest CSP. **(C)** CSP plot between 0 mM and 30 mM Ca^{2+}. Metal-binding sites A and B and residues near site A (Gln14, Leu15, Gly16, and Val17) are high-lighted in yellow, purple, and sky-blue, respectively.

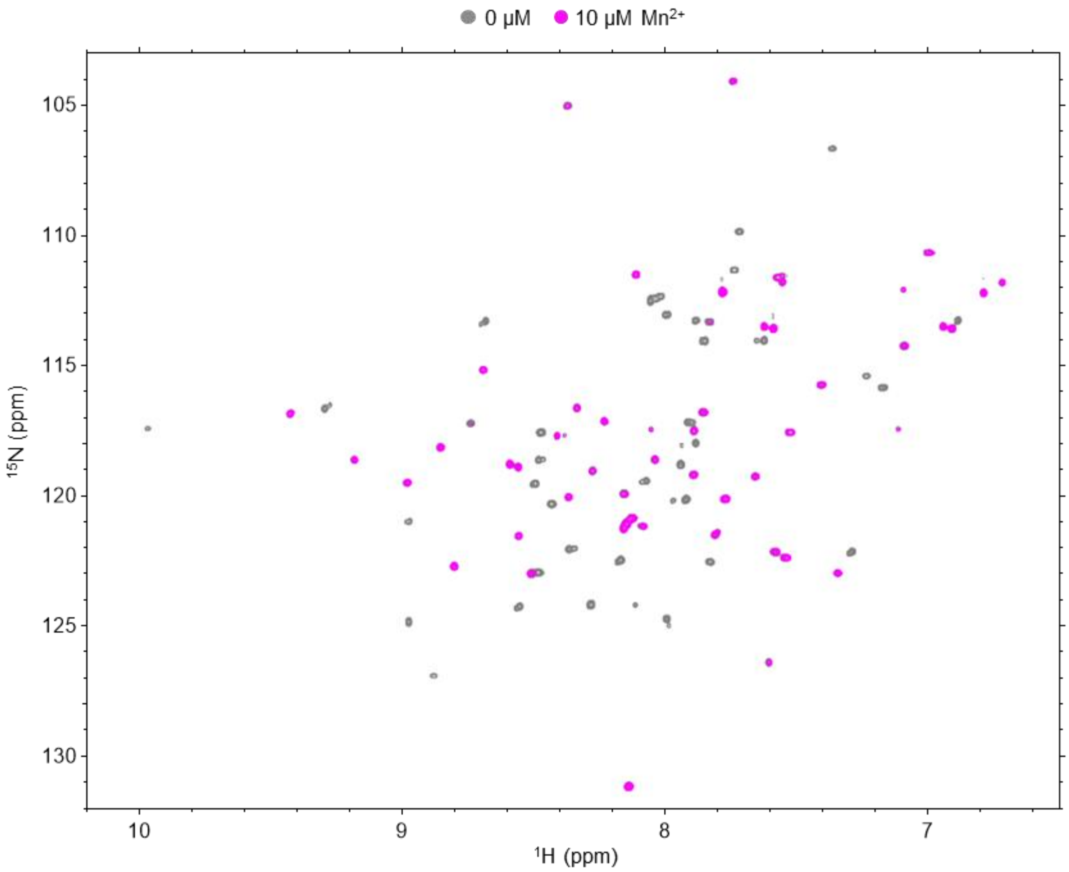

Figure S2. NMR Mn^{2+} titration of holo-*Ec*ACP. Overlaid ^{1}H-^{15}N HSQC spectra of holo-*Ec*ACP in the presence of 0 µM (gray) and 10 µM (magenta) Mn^{2+}.

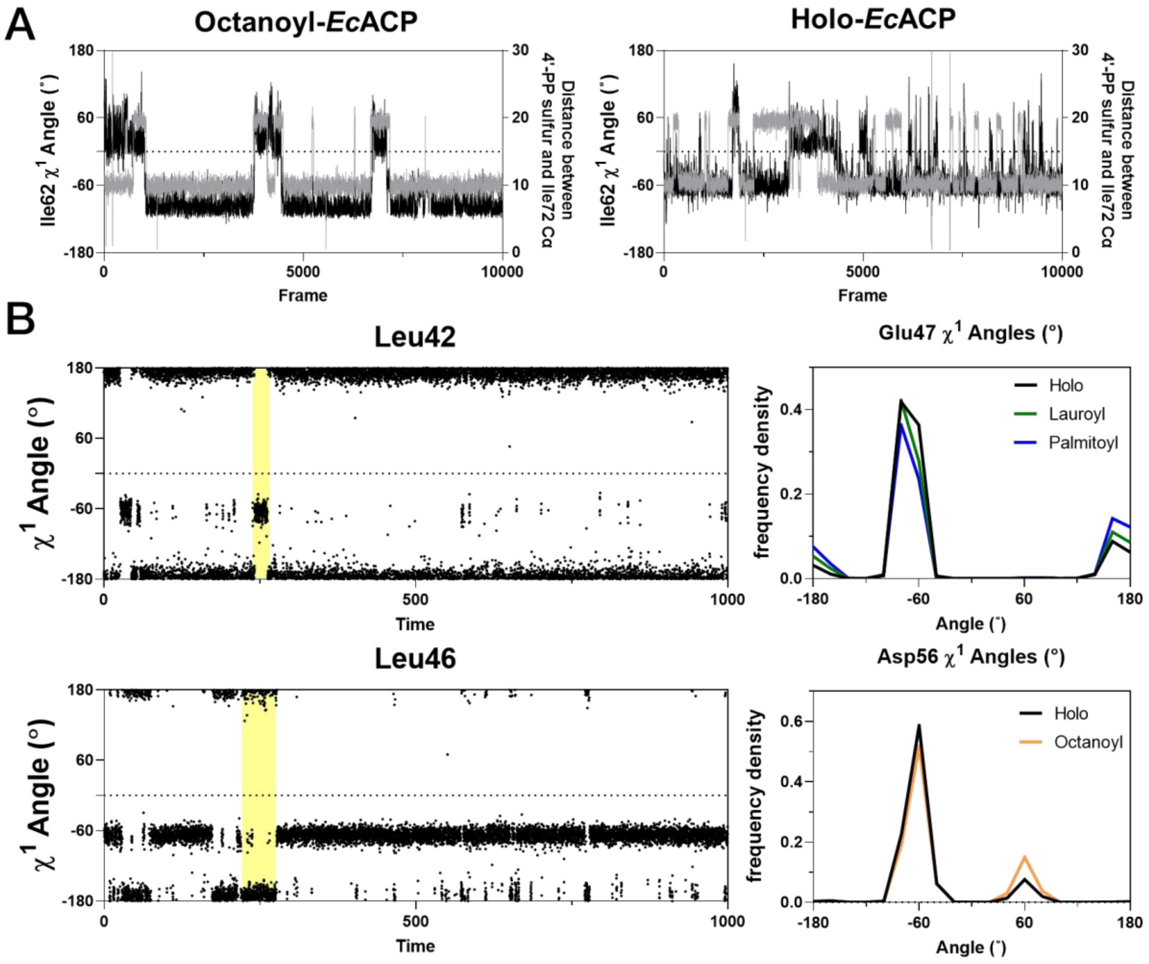

Figure S3. Numerical data of *Ec*ACP from MD siumlations. (A) Ile62 χ1 angle distribution (grey) and distance between sulfur on phosphopantetheine group and center of mass (COM) (black). (B) Variation of χ1 torsion angle of Leu42 and Leu46 in palmitoyl-ACP during 1 μs MD simulations (left). χ1 angle of Glu47 and Asp56 shown in various form of *Ec*ACP (right). The yellow shading indicates that two leucine residues perform the gating function simultaneously.

Supplementary References

1. Lee, W., J.L. Stark, and J.L. Markley, *PONDEROSA-C/S: client-server based software package for automated protein 3D structure determination.* J Biomol NMR, 2014. **60**(2-3): p. 73-5.
2. Lee, W., et al., *Integrative NMR for biomolecular research.* J Biomol NMR, 2016. **64**(4): p. 307-332.
3. Bhattacharya, A., R. Tejero, and G.T. Montelione, *Evaluating protein structures determined by structural genomics consortia.* Proteins, 2007. **66**(4): p. 778-95.
